# Benchmarking DNA Extraction Methods for Phylogenomic Analysis of Sub-Antarctic *Rhodococcus* and *Williamsia* Species

**DOI:** 10.3390/microorganisms9061253

**Published:** 2021-06-09

**Authors:** Akhikun Nahar, Anthony L. Baker, David S. Nichols, John P. Bowman, Margaret L. Britz

**Affiliations:** 1 Tasmanian Institute of Agriculture, University of Tasmania, Hobart, TAS 7005, Australia; Anthony.baker@outlook.com.au (A.L.B.); john.bowman@utas.edu.au (J.P.B.); 2 Central Science Laboratory, Division of Research, University of Tasmania, Hobart, TAS 7005, Australia; d.nichols@utas.edu.au

**Keywords:** Actinobacteria, *Rhodococcus erythropolis*, *Rhodococcus qingshengii*, *Williamsia*, DNA extraction, next-generation sequencing, mycolic acids, ANI, psychrotrophic

## Abstract

Bacteria containing mycolic acids in their cell envelope are often recalcitrant to cell lysis, so extracting DNA of sufficient quality for third-generation sequencing and high-fidelity genome assembly requires optimization, even when using commercial kits with protocols for hard-to-lyse bacteria. We benchmarked three spin-column-based kits against a classical DNA extraction method employing lysozyme, proteinase K and SDS for six lysozyme-resistant, sub-Antarctic strains of *Corynebaceriales*. Prior cultivation in broths containing glycine at highly growth-inhibitory concentrations (4.0–4.5%) improved cell lysis using both classical and kit methods. The classical method produced DNA with average fragment sizes of 27–59 Kbp and tight fragment size ranges, meeting quality standards for genome sequencing, assembly and phylogenomic analyses. By 16S rRNA gene sequencing, we classified two strains as *Williamsia* and four strains as *Rhodococcus* species. Pairwise comparison of average nucleotide identity (ANI) and alignment fraction (AF), plus genome clustering analysis, confirmed *Rhodococcus* sp. 1163 and 1168 and *Williamsia* sp. 1135 and 1138 as novel species. Phylogenetic, lipidomic and biochemical analyses classified psychrotrophic strains 1139 and 1159 as *R. qingshengii* and *R. erythropolis*, respectively, using ANI similarity of >98% and AF >60% for species delineation. On this basis, some members of the *R. erythropolis* genome cluster groups, including strains currently named as *R. enclensis*, *R. baikonurensis*, *R. opacus* and *R. rhodochrous*, would be reclassified either as *R. erythropolis* or *R. qingshengii*.

## 1. Introduction

Contemporary genome sequencing technologies, described as third- and next-generation sequencing (such as single molecule real-time (SMRT) sequencing) facilitate the assembly of complex genomes by generating read lengths of >10 kb [1]. Increasingly, these technologies require high-molecular weight genomic DNA (gDNA) templates. High-quality and high-molecular weight DNA is also recommended for short reading length using next-generation sequencing technologies [2,3]. However, some Gram-positive bacterial cells are very difficult to disrupt, particularly species of Actinobacteria in the order *Corynebacteriales*, which includes the genera *Mycobacterium*, *Corynebacterium*, *Rhodococcus*, *Williamsia*, *Nocardia* and *Gordonia*. Species within these genera are of biotechnological interest due to their broad catalytic activity in degrading toxic and persistent pollutants [4,5,6], synthesis of storage compounds with potential as biofuels (including triacylglycerols) or specialty chemicals [7,8,9] and synthesis of bioactive compounds and enzymes of pharmacological interest or with industrial applications [10,11]. The *Corynebacteriales* are highly or partially resistant to lysozyme due to their complex, lipid-rich cell envelopes which include mycolic acids covalently bound to arabinogalactan thence peptidoglycan [12]. gDNA extraction often requires prolonged enzymatic digestion, use of several enzymes and sometimes other chemical or mechanical interventions [13], which may result in fragmented DNA. Different commercial kits are available to extract high-quality gDNA with specific instructions for ‘hard-to-lyse’ bacteria, replacing conventional phenol chloroform extraction methods developed in the 1960s [14] and modified subsequently for different organisms [15]. However, variations in DNA yield and quality can occur [16], often necessitating optimizing conditions for lysis and DNA extraction for different species [17,18].

After failing to extract gDNA suitable for shotgun genome sequencing from six lysozyme-resistant sub-Antarctic *Corynebacteriales* strains, including testing various enzymatic and chemical treatments in combination with kit-based methods and alternative purification protocols, we investigated whether prior culture in glycine-supplemented broth would improve cell lysis. Growth in glycine-supplemented media is known to reduce peptidoglycan cross-linking so the structure is more amenable to lysozyme digestion [19] and has been employed previously to improve spheroplast formation in *Mycobacterium* spp. [20] and as a pre-treatment for improving DNA uptake by electroporation in *Corynebacterium glutamicum* [21].

We report the impact of prior growth conditions on the quality of gDNA extracted from the six environmental isolates of *Corynebacteriales*, as evaluated from fragment size and size distribution, using a modified, optimized classical extraction method [14] which was later compared with three commercial gDNA extraction kits. The gDNA prepared using the classical method met the quality criteria for using genome data for taxonomic purposes in prokaryotes [22] and genome sequencing confirmed the preliminary taxonomic delineation obtained by 16S rRNA gene sequencing. While two *Williamsia* and two *Rhodococcus* strains were deemed novel species, based on ANI and phylogenomic analyses, two sub-Antarctic strains were classified as *R. qingshengii* (1139) and *R. erythropolis* (1159). There is increasing evidence that several validly published species of *Rhodococcus* in the ‘*erythropolis*’ mRNA sequence cluster [23] are later heterotypic synonyms, including species designated as *R. degradans*, *R. enclensis* and *R. jialingiae* [24,25,26]. Consequently, the biochemical and physiological properties of strains 1139 and 1159 were determined to document the properties of these psychrotrophic strains, including modelling the lowest temperature of growth. Species delineation points based on ANI and AF analyses for *Rhodococcus* in the ‘*erythropolis*’ genome clusters are discussed.

## 2. Materials and Methods

### 2.1. Bacterial Strains and Culture Conditions

The six strains from the University of Tasmania (Australia) sub-Antarctic culture collection (four identified as *Rhodococcus* spp., 1139, 1159, 1163 and 1168, and two as *Williamsia* spp., 1135 and 1138) [27,28,29] were cultured in a minimal salts media (MSM) consisting of a basal salts solution (BSS) including 7 g/L K_2_HPO_4_, 2 g/L KH_2_PO_4_, 0.25 g/L sea salt (Instant Ocean, Blacksburg, VA, USA), 2 g/L nitrogen source, 10 g/L carbon source, 0.01 g/L FeSO_4_ and 10 mg/L thiamine hydrochloride (Sigma-Aldrich, Castle Hill, NSW, Australia). For routine culture of bacterial strains and the control strain *R. corynebacteriodes*, the nitrogen source used was NH_4_H_2_PO_4_ while the carbon source was *D*-fructose (MSM-F). The sub-Antarctic strains are available in the Belgium BCCM/LMG collection: 1139 = LMG 32064; 1159 = LMG 32063.

To determine the impact of glycine on bacterial growth, 300 µL MSM-F (final volume) supplemented with up to 6% (*w*/*v*) glycine (MSM-G) was inoculated with mid-exponential phase cells to an initial optical density at 600 nm (OD_600_) of 0.1 in a 100-well Bioscreen plate. OD_600_ was recorded every 2 h for up to 18 days at 25 °C using Bioscreen-C instrumentation (Oy Growth Curves Ab Ltd., Helsinki, Finland) for triplicate cultures. Maximum specific growth rates (µ_max_) were calculated using Monod equations [30] and standard deviations calculated.

To extract gDNA for sequencing, strains were initially cultured in 30 mL MSM-F at 25 °C at 200 rpm then mid-exponential phase cells collected by centrifugation at 10,000 rcf for 5 min. Cells were resuspended in 50 mL MSM-G with varying concentrations of MSM-G (4.5% for *Williamsia* sp. 1138 and the four *Rhodococcus* spp.; 4.0% for *Williamsia* sp. 1135) with incubation continued until the initial OD_600_ doubled. *Escherichia coli* strain M13 was used as a positive control for gDNA extraction using the kits and was cultured in Nutrient Broth (Oxoid, Thermo Fisher Scientific Australia Pty Ltd., Scoresby, VIC, Australia).

### 2.2. Benchmarking Extraction and Purification of gDNA

Due to its demonstrated recalcitrance to lysis, *Williamsia* sp. strain 1138 was chosen to determine the effects on the quality of extracted DNA following prior culture in glycine-supplemented media (0, 2.0 and 4.5% MSM-G). Three commercial kits were used in this study (Method 1: ISOLATE II Genomic DNA Kit (Bioline, Alexandria, NSW, Australia); Method 2: Ultraclean Microbial DNA Isolation Kit (MO BIO Laboratories, Inc., Carlsbad, CA, USA); and Method 3: QIAamp^®^ DNA Mini Kit (QIAGEN GmbH, Hilden, Germany)) and a classical method (Method 4).

*Williamsia* sp. 1138 and *E. coli* cells were collected from 50 mL of culture by centrifugation (10,000 rcf for 5 min) and cell pellets resuspended in 0.01 M Tris buffer (pH 8) to concentrate to an OD_600_ of 4.5. Triplicate aliquots of 1 mL were transferred into 1.5 mL microcentrifuge tubes for each treatment and growth condition, then cells were collected by centrifugation as described above and resuspended in buffers as specified in each protocol. DNA was extracted according to instruction manuals for hard-to-lyse bacteria except for elution steps, when DNA was eluted in smaller volumes of elution buffer to increase final DNA concentrations. Genomic DNA was extracted from *E. coli* according to the instruction manuals for bacteria.

For the classical extraction method (Method 4), based on approaches originally described by Marmur [14], cells were resuspended in 180 µL 0.05 M Tris/EDTA buffer, pH 8, supplemented with 20% sucrose. Lysozyme (200 µg/mL) (Sigma-Aldrich, Castle Hill, NSW, Australia) was added followed by incubation at 37 °C for 2 h (with 4–5 inversions during incubation). Lysozyme-treated cells were collected by centrifugation at 15,000 rcf for 5 min and resuspended into 600 µL Tris/EDTA buffer containing 60 µg/mL proteinase K, 100 μg/mL DNase-free RNase and 1% SDS (all reagents from Sigma-Aldrich, Castle Hill, NSW, Australia), then incubated at 55 °C for 1 h. Subsequently, 600 µL of chloroform/*iso*-amyl alcohol (24/1, *v*/*v*) was mixed with the viscous solution, then the emulsion separated by centrifugation at 15,000 rcf for 30 min. The top layer was carefully collected into a new tube by pipetting and the extraction repeated twice. After the final collection of the aqueous phase, two volumes of ice-cold ethanol and ammonium acetate (0.3 M final concentration from a 3 M solution) were added to precipitate DNA. After 5 min on ice, the precipitate was collected (12,000 rcf, 4 °C, 10 min) then the supernatant carefully discarded without disturbing the DNA pellet. The DNA pellet was washed twice with 500 µL of 70% (*v*/*v*) ice-cold ethanol to remove the salts, centrifuged for 1 min at 12,000 rcf, then the pellet was air dried for 10 min prior to resuspending into 50 µL of 0.01 M Tris buffer (pH 8).

### 2.3. Quantity, Fragment Size and Purity of Extracted DNA

Preliminarily, DNA concentration was estimated from absorbance at 260 nm with protein contamination being determined from the A_260_/A_280_ and other organic contamination determined by A_260_/A_230_ ratio (NanoDrop 8000 Spectrophotometer, Thermo Fisher Scientific Australia Pty Ltd., Scoresby, VIC, Australia).

Quantity and fragment size of extracted DNA was assessed using a DNA Fragment Analyser (Advanced Analytical Technologies, Inc, Orangeburg, NY, USA) and the PROSize^®^ 2.0 software was used to analyse data, including imaging each electrophoretogram. A DNF-487-33-SS Genomic DNA kit (Advanced Analytical Technologies, Inc., Orangeburg, NY, USA) was used as internal standard to ascertain size and quantity of DNA. The lower marker (LM) was set to 1 base pair (bp) and 10,000 bp was set as the threshold for DNA quality number (GQN). The PROSize^®^ 2.0 software scores the GQN value 0–10 based on the percentage of DNA above the set threshold. 

### 2.4. gDNA Sequencing and Quality Assessment

gDNA was prepared for all the strains from 50 mL cultures, grown in 4 or 4.5% MSM-G, as described in Section 2.2, Method 4, with slight variation. After lysozyme treatment, cells collected from strains *Williamsia* sp. 1135, *Williamsia* sp. 1138, *Rhodococcus* sp. 1139 and *Rhodococcus* sp. 1159 were incubated with proteinase K for 1 h to achieve complete lysis (visual clearing of turbidity), and for 3–4 h for strains *Rhodococcus* sp. 1163 and *Rhodococcus* sp. 1168. Extracted gDNA samples were sent to Macrogen Inc (Seoul, Republic of South Korea) for MiSeq Illumina sequencing, which included quality assessment. A 670-bp size-based TruSeq Nano DNA (550) Library was prepared and Illumina SBS technology used to generate 2 × 300-bp paired-end reads. MiSeq Control Software v2.2 (MCS) was used to generate raw data and Real Time Analysis software (v1.18) was used for base calling. To access the sequencing data quality, the base calling accuracy was measured by phred quality score (Q score).

### 2.5. 16S rRNA Gene Sequencing and Preliminary Phylogenetic Analysis

The ISOLATE II Genomic DNA Kit (Bioline, Alexandria, NSW, Australia) was used to extract DNA from mid-exponential phase cultures (without glycine supplementation) according to the manufacturer’s directions for hard-to-lyse bacteria. The universal primers 27F and 16S-1492R (Sigma-Aldrich, Castle Hill, NSW, Australia) were used to amplify a 1465-bp segment of the 16S rRNA gene. PCR products were sequenced by Macrogen Inc. Sequences were analysed using the nucleotide BLAST function of the National Centre for Biotechnology Information (NCBI) and the closest match was determined to assign probable identities. Multiple sequence alignment option CLUSTALW (https://www.ebi.ac.uk/Tools/msa/clustalo/) was used to align the 16S rRNA sequences and a neighbour-joining phylogenetic tree was performed using DNADIST v3.5c program of BioEdit software (v7.2.5) [31] to determine their position within the genus.

### 2.6. Genome Sequence Analyses to Identify Rhodococcus Species

The sequenced genomes of all six sub-Antarctic strains were examined using tools available in the Integrated Microbial Genomics and Microbiomes (IMG/M) database (https://img.jgi.doe.gov/cgi-bin/mer/main.cgi, accessed most recently in May 2021) [32] for pairwise ANI and genome-clustering analyses plus phylogenetic tree construction using hierarchical clustering based on pfam and COG functions. IMG/M genome identification numbers are in order of our strain numbers 1135–1168: 2724679737, 2775507282, 2724679739, 2816332322, 2724679741 and 2724679742. Pairwise ANI comparisons were made using the NCBI reference genome for *R. erythropolis* (CCM 2595), a recently sequenced genome of *R. qingshengii* TUHH-12 [33], and type strains of both species, *R. erythropolis* NBRC 15567 (= JCM 3201 = ATCC 4277) and *R. qingshengii* (JCM 15477 and Djl-6-2) (from the List of Prokaryotic names with Standing in Nomenclature, https://lpsn.dsmz.de/, accessed January 2021) [34]. Genomes of other strains which were detected as similar to the query isolates by 16S rRNA gene sequencing were selected for comparison from the *Rhodococcus* species listed on IMG/M. As the whole genomes of some species that were most closely related to strains *Rhodococcus* sp. 1163 and 1168 (based on 16S rRNA gene sequences) were not available when this research was undertaken, a neighbour-joining phylogenetic tree based on the *alkB* gene was prepared using MEGA7 software [35]. Multiple sequence alignment option MUSCLE in the MEGA7 software was used to align the *alkB* gene sequences. AlkB is a key protein in the alkane-degrading pathway and the gene is used as a phylogenetic marker to differentiate closely related *Rhodococcus* species [36]. 

### 2.7. Biochemical Characterization of Strains 1139 and 1159

#### 2.7.1. Enzymatic Tests to Characterize Bacterial Strains

To detect the enzymatic activities of bacterial strains, the semi-quantitative micro-method API ZYM (bioMérieux, North Ryde, NSW, Australia) was used, where each strip contained 20 cupules (19 enzymatic substrates and 1 control). Bacterial cells grown in MSM-F were collected by centrifugation at 10,000 rcf for 5 min, washed with sterile phosphate-buffered saline (PBS) and resuspended in PBS with OD_600_ adjusted to 0.5. Optical density was measured using an Ultraspec spectrophotometer (BMG Labtech Pty. Ltd., Mornington, Australia). Aliquots of 65 µL of this suspension were transferred aseptically into each cupule of the supplied API strip, incubated overnight at 25 °C and reagents were added according to manufacturer’s directions. Results were recorded in values ranging from 0 to 5 according to the degree of color development, where 0 corresponds to a negative reaction and 5 corresponds to maximum intensity.

#### 2.7.2. Growth on Different Carbon and Nitrogen Sources and Impact of Phosphate Concentration on Growth

To compare growth rates on different carbon sources, the *Rhodococcus* strains 1139 and 1159 were grown initially in 30 mL of MSM-F at 25 °C, 200 rpm, to mid-log phase. The OD_600_ was measured, then cells collected by centrifugation at 10,000 rcf for 5 min. After decanting the supernatant, cells were washed twice with BSS and incubated for 24 h in the same solution at 25 °C and 200 rpm agitation. The cell suspensions were centrifuged again, and pellets were resuspended in a volume of BSS to concentrate cells to an OD_600_ of approximately 10. This suspension was then used to inoculate 297 µL MSM containing 1% (*w*/*v*) of different carbon sources to adjust the final volume to 300 µL with an initial OD_600_ of 0.1 in a 100 well Bioscreen plate with three replicates for each test. The Bioscreen C instrument was set at 25 °C with continuous medium amplitude shaking. A grey filter with 600 nm wavelength was selected to record the OD at 2-h intervals for up to 12 days. The maximum readable OD_600_ with this system was 3.5.

To compare bacterial growth on different nitrogen sources, MSM-F was prepared with 0.2% (*w*/*v*) of different organic and inorganic nitrogen sources, plus combinations of these. Double-strength BSS was sterilized by autoclaving and filter-sterilized carbon and nitrogen sources were added from 20 (*w*/*v*) and 2% (*w*/*v*) stock solutions, respectively, with the final volume adjusted with sterile distilled water to produce single-strength MSM. Bacterial strains were inoculated, and growth was observed using the automated Bioscreen system as described above. When the nitrogen source in MSM-F broth, NH_4_H_2_PO_4_, was replaced by alternative nitrogen sources, the concentration of phosphate ion (6.8 g/L) was concurrently reduced by 25% (to 5.2 g/L). To evaluate the impact of phosphate concentration on growth, a second series of MSM-F media with the different nitrogen sources was prepared by adjusting the composition of BSS to contain 9.41 g/L K_2_HPO_4_ and 2.52 g/L KH_2_PO_4_ (6.8 g/L phosphate ion), including when NH_4_H_2_PO_4_ was used as nitrogen source (total phosphate ion 8.5 g/L, or a 25% increase above MSM-F). This second set of media is described as ‘phosphate-adjusted’.

#### 2.7.3. Salt Tolerance Testing

To evaluate salt tolerance, double-strength BSS-NH_4_H_2_PO_4_ was supplemented with 0 to 15% NaCl from a 25% (*w*/*v*) filter sterilized NaCl stock solution. *D*-fructose, thiamine hydrochloride and FeSO_4_ were supplemented as for regular MSM, and then the volume adjusted to single-strength MSM with sterile distilled water. The strains were inoculated and cultured using the Bioscreen system as described above.

#### 2.7.4. Determining Optimum Temperature for Growth in MSM-F Broth

Growth rates for temperatures in the range between 0 and 45 °C were determined in shake-flask cultures. To prepare starter cultures, a single colony was picked from an NA streak-plate and inoculated into 30 mL MSM-F broth in 100 mL conical flasks and cultured at 25 °C, 200 rpm. At mid-log phase, the starter was sub-cultured into 100 mL MSM-F broth to give an initial OD_600_ of 0.06. Optical density was recorded periodically during incubation against the medium blank for up to 4 months depending on temperature. Cultures were diluted 10- or 100-fold in the same medium when the OD_600_ reached above 0.9 (within the linear range of turbidity for the spectrophotometer). All tests were performed in triplicate.

#### 2.7.5. Modelling Minimum and Maximum Growth Temperatures

Growth rate (µ_max_) was calculated from the natural logarithm of OD_600_ for MSM-F-grown cells: µ_max_ = (Ln N2 − Ln N1)/(t2 − t1), where N = cell concentration and t = time (30). Generation time (GT) (h) was calculated by dividing the natural logarithm of 2 by µ_max_ (h^−1^): GT = Ln2/µ_max_. Generation times at different temperature were used to fit the nonlinear model [37] below using the function nlsLM in the library minpack.lm using the software program R version 3.3 [38]. The experimentally determined and model-predicted values were plotted against temperature. The fitted function was:

$$\sqrt{r}=b\times (T-T_{\min})\times (1-\exp(c\times (T-T_{\max}))$$

where r = observed growth rate, T = temperature (°C) and b, c, T_min_ and T_max_ are regression coefficients. The T_min_ and T_max_ represent the temperatures at which growth is zero. The model was fitted to the square root of the growth rate to homogenize variance.

#### 2.7.6. Triacylglycerol (TAG) Analysis

Positive ESI technique was used to identify TAG with UPLC–MS/MS. A Waters Acquity UPLC BEH C18 column (2.1 mm × 100 mm × 1.7 µm) was used. The UPLC solvent program consisted of solvent A 100 mM ammonium acetate (pH 5), solvent B acetonitrile and solvent C methanol:hexane (8:2, *v*/*v*). The solvent gradient was: 100% A/B (2:8, *v*/*v*) from 0 min to 100% B at 2 min; followed by a linear gradient to 100% C at 8 min, which was held for 14 min before returning to initial conditions. The total flow was 0.5 mL/min for 2 min followed by an immediate ramp to 0.6 mL/min. A post-column infusion of 5% (*v*/*v*) ammonium hydroxide solution was employed at a rate of 3 µL/min to induce the formation of [M+NH_4_]^+^ molecular species. TAG molecular species were detected in full scan mode over the mass range (*m*/*z*) 100 to 1050 at three simultaneous cone voltages (20, 45 and 70 V). The higher cone voltages were used to induce in-source fragmentation for partial identification of TAG acyl residues [39].

#### 2.7.7. Fatty Acid Methyl Ester (FAME) Analysis by GC–MS

FAMEs were analysed by gas chromatography–mass spectrometry (GC–MS) consisting of a Varian 3800 GC and a Bruker 300 triple quadrupole MS fitted with a 30 m × 0.25 mm Agilent VF-5MS column (0.25 µm film thickness, bound fused silica, Agilent Technologies, Santa Clara, CA, USA) and using helium (1.3 mL/min) as the carrier gas. A 1 µL portion of sample was injected with a 30:1 split ratio. The injection port was kept at 290 °C. The oven temperature was initially 50 °C for 1 min and then programmed to heat at a rate of 30 °C/min to reach 150 °C, then 2 °C/min to reach 250 °C and after that, 5 °C/min to reach the final temperature 320 °C for 5 min. Star software was used to control the operation of the GC–MS. Full-scan MS spectra in the range of (*m*/*z*) 35 to 450 was obtained. Individual FAMEs were identified by comparison of retention times and mass spectra with those of standard FAME (NIST/EPA/NIH Mass Spectral Library 2017). Relative amounts of each FAME were calculated as a percentage of the total amount (peak area) detected.

### 2.8. Analysis of Mycolic Acids (MA) 

Bound and unbound MA were identified by UPLC–triple quadruple mass spectrometry (UPLC–MS/MS) in negative electrospray ionization (ESI) mode using a Waters Acquity H-Class UPLC instrument coupled to a Waters Xevo TQ MS/MS (Waters Corporation, Milford, MA, USA). A Waters Acquity UPLC BEH C18 column (2.1 mm × 100 mm × 1.7 µm) was used (Waters Corporation). The solvents for UPLC consisted of solvent A, acetonitrile containing 1 mM acetic acid, and solvent B, isopropanol:hexane (8:2, *v*/*v*). Initial conditions were 100% solvent A for 1 min, before a gradient elution to 100% solvent B over 14 min, which was held for 5 min before returning to initial conditions and equilibrating for 3 min. Total flow was 0.3 mL/min. For MA analysis the electrospray needle was set at 2.8 kV. The ion source temperature was 130 °C, the desolvation gas was N_2_ at 950 L/h, the cone gas flow was 100 L/h and the desolvation temperature was 450 °C. MA molecular species were detected in full-scan mode experiments over the mass range (*m*/*z*) 400 to 1000 with a cone voltage of 60 V. MS/MS product scan experiments were conducted from specific [M − H]^−^ precursor molecules over an appropriate product ion range using a cone voltage of 60 V and collision energy of 45 V. Data were processed using MassLynx software (version 4.1) (Waters Corporation).

## 3. Results

### 3.1. Impacts of Glycine on Bacterial Growth 

**Figure 1:**
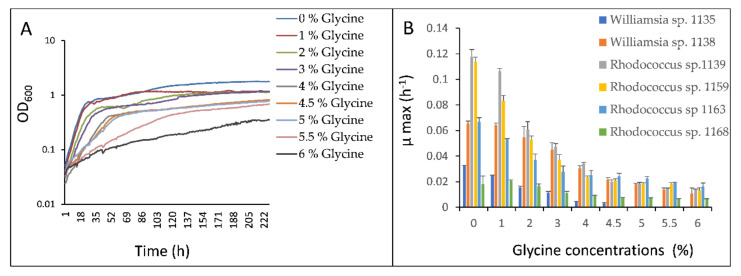
Impact of glycine on growth of six sub-Antarctic strains. (**A**) An example of growth kinetics of strain *Williamsia* sp. 1138 in MSM-F broth supplemented with different concentrations of glycine; (**B**) means and standard deviations of maximum specific growth rates (µ_max_) for six strains cultured in MSM–glycine broths.

The six sub-Antarctic strains demonstrated different sensitivity to growth inhibition by glycine when cultured in MSM-F broth (Figure 1). The majority were not impacted greatly by 1% glycine, with µ_max_ values at 75–115% of the control. Although four of the strains demonstrated approximately 50% inhibition at 2% glycine, *Williamsia* sp. 1138 and *Rhodococcus* sp. 1168 only showed similar inhibition when MSM-F broth was supplemented with 4% glycine. *Williamsia* sp. 1135 was more sensitive to growth inhibition by glycine, showing strong inhibition at 4% and failure to grow at 5–6% glycine. All the other strains grew in glycine concentrations up to 6%, albeit at highly reduced µ_max_ values.

### 3.2. Quantity and Purity of Extracted DNA

Fragment Analyser data indicated that all the column-based kits extracted high-molecular weight gDNA from *E. coli*, with an upper fragment size of 39 to 52 Kbp for the different kits (Table 1). However, the smallest fragment sizes (1–7 Kbp) varied with the kits: a GQN number of 6.5, corresponding to the broadest fragment size range and lowest average fragment size, was obtained using the Isolate II Genomic DNA kit (Method 1), while the highest GQN number of 9.2 was found from the QIAAMP Mini kit (Method 3) (Table 1). *E. coli* gDNA yields were higher than seen for the Gram-positive *Williamsia* sp. 1138 (Table 1). These data validate the efficacy of all the kits with the *E. coli* control.

**Figure 2:**
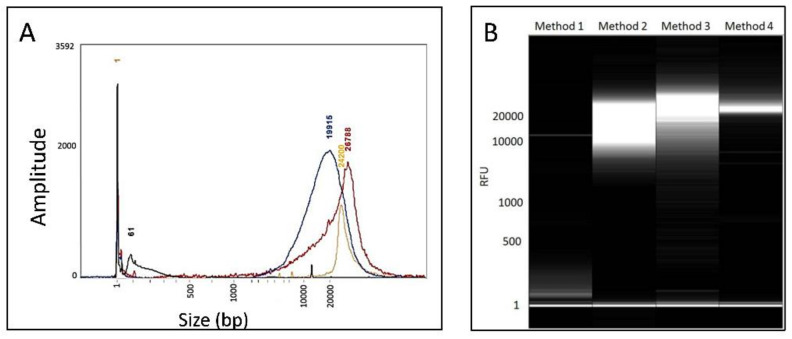
DNA fragment size analyses for four extraction methods of *Williamsia* sp. 1138 cells cultured in 4.5% MSM-G. (**A**) Amplitude readout from the Fragment Analyser and (**B**) images generated from the automated capillary electrophoresis system of the Fragment Analyser with size standards (bp) marked. Method 1: Isolate II Genomic DNA kit (Bioline) (_ peaks at 61 and 12,000 bp; Method 2: Ultraclean Microbial DNA Isolation kit (MO BIO) (_ peak at 19,915 bp); Method 3: QIAAMP Mini kit (Qiagen) (_ peak at 26,788 bp); Method 4: Classical extraction method (_ peak at 24,200 bp).

Prior culture of *Williamsia* sp. 1138 in glycine-supplemented broth improved both the yield (DNA concentration from the same starting biomass in each test) and GQN for all four methods. Excepting Method 3, prior culture in 4.5% MSM-G improved both gDNA yield and GQN relative to culture in 0 and 2% MSM-G. However, Method 1 produced fragmented DNA from cells cultured in 0, 2 and 4.5% MSM-G under the experimental conditions used (Table 1, Figure 2). The highest concentration of gDNA was isolated using Method 3, although the maximum DNA integrity (assessed in terms of average fragment size and tightest fragment size range, and a GQN of 9) was obtained by Method 4 for cells cultured in 4.5% MSM-G, as demonstrated by tight banding seen on gel images (Figure 2). Acceptable A_260_/A_280_ and A_260_/A_230_ ratios were obtained for most of the kit extracts and for gDNA obtained by Method 4 for 4.5% glycine-grown cells (Table 1).

### 3.3. DNA Sequencing and Quality Score of Sequenced DNA

The classical gDNA extraction method was subsequently applied to all six sub-Antarctic strains using 50 mL cultures of MSM-G with 4.0 or 4.5% glycine and DNA submitted for shotgun sequencing. The quality of the DNA was high (as assessed from high average DNA fragment size, GQN number and absorbance 260/280 and 260/230 ratios) (Table 2). A total of 1.9, 1.8, 1.7, 1.7, 1.8 and 1.9 Gb read bases with over 60% Q30 score were obtained for Williamsia spp. 1135 and 1138 and Rhodococcus spp. 1139, 1159, 1163 and 1168, respectively. Raw data from Macrogen Inc was assembled using ABySS software [27,28,29] and submitted to DDBJ/EMBL/GenBank under the accession numbers: MJEI00000000; MJEJ00000000; LWHK00000000; MJVD00000000; MKKX00000000; and MKKY00000000.

### 3.4. Preliminary Strain Identification Based on Sequencing 16S rRNA Gene PCR Amplicons 

**Figure 3:**
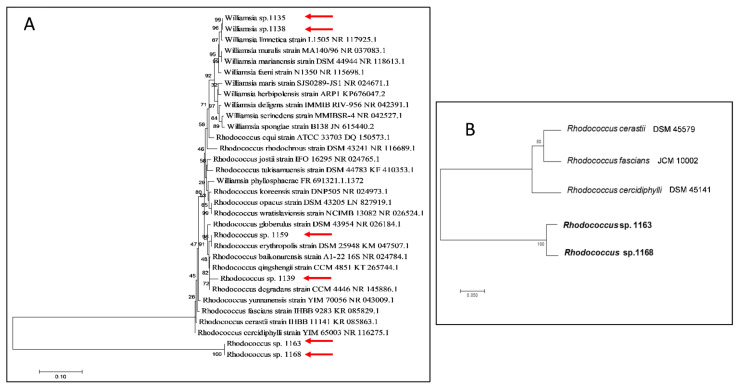
Neighbour-joining phylogenetic trees based on (**A**) BLAST analysis of 16S rRNA gene sequences obtained by PCR amplification of DNA extracted from six sub-Antarctic strains and (**B**) alkane 1-monooxygenase gene (*alkB*) gene sequences from whole genome sequences of *Williamsia* spp. 1135 and 1138 and *Rhodococcus* spp. 1139, 1159, 1163 and 1168. Bootstrap values shown at branch nodes were based on 1000 re-samplings and the *alkB* gene sequences in (**B**) are from published genomes of the type strains for the species selected. The sub-Antarctic strains are marked with a red arrow in panel (**A**).

The BLAST search results based on the 16S rRNA gene sequences indicated that strains 1135 and 1138 belonged to the genus *Williamsia,* while the other strains belonged to the genus *Rhodococcus* (Figure 3A). Known species of *Williamsia* formed two distinct clades with strains 1135 and 1138 demonstrating 99% similarity with the clade containing species of *W. muralis*, *W. faeni*, *W. marianensis* and *W. limnetica*. While most closely related to *W. limnetica*, strains 1135 and 1138 were distinct from this species and from each other based on the 16S rRNA neighbour-joining tree (Figure 3A). The sequence of the 16S rRNA PCR products from strains 1139 and 1159 showed 99 and 100% similarity, respectively, with *R. erythropolis* R138 and clustered with a group of *Rhodococcus* species including *R. erythropolis*, *R. qingshengii*, *R. baikonurensis* and *R*. *degradans*. Strain 1163 had 99% similarity with *R. fascians* strain IHBB 9283 and *R. yunnanensis* strain R-36475. Strain 1168 had 99% similarity with *R. fascians* IHBB 9283, *R. cercidiphylli* EB37, *R*. *cerastii* BZ22 and *R. yunnanensis* IHBB 9867. *Rhodococcus* strains 1163 and 1168 each formed separate clades in 16S rRNA gene neighbour-joining trees. Assigning strain identity based on 16S rRNA gene sequence similarity was not possible given that several different species were closely clustered, although it was clear that the two sub-Antarctic *Williamsia* and two *Rhodococcus* species were distinct from their nearest neighbours in the phylogram. Sequences for the *alkB* gene from type strains of *R. cerastii*, *R. fascians* and *R. cercidiphylli* were compared with this gene in *Rhodococcus* sp. 1163 and 1168 (Figure 3B), which confirmed that these sub-Antarctic strains formed a separate clade to the species most closely related by 16S rRNA gene sequencing.

### 3.5. Taxonomy of Rhodococcus Isolates Based on Analysis of Sequenced Genomes

Figure S1 shows a phylogram based on pfam hierarchical clustering for all *Rhodococcus* genomes in the IMG/M database (accessed March 2020). Strain 1139 clustered with species named as *R. erythropolis*, *R. qingshengii* and *R. baikonurensis*, and 1159 clustered with strains named *R. erythropolis*, *R. qingshengii* and *R. enclensis*. Strains 1163 and 1168 clustered with unclassified strains and the closest neighbours were two Antarctic soil isolates, *Rhodococcus* spp. PAMC 28705 and PAMC 28. The latter was confirmed when a phylogenetic tree was constructed (NCBI Genome BLAST, Taxonomy Browser) for all unclassified species of *Rhodococcus* (Figure S2), which indicated that strains 1163 and 1168 were distinct from each other and likely represented novel species of *Rhodococcus*.

Pairwise ANI and AF calculations were performed to compare the sub-Antarctic rhodococci with type strains of *R. erythropolis* and *R. qingshengii*, as shown in the Table 3 (Part A), and the Antarctic soil isolates for strain 1163 and 1168 (Part B). Using an ANI species delimitation of ≥ 96.5% and AF of ≥ 60% [40], strain 1139 would be classified as *R. qingshengii* and 1159 as *R. erythropolis*. Strains 1163 and 1168 showed < 96.5% ANI and on that basis would be considered distinct from their nearest neighbours, the Antarctic soil strains, although AF was >60%, possibly indicating a close relationship between these species.

Using the IMG/M ANI genome-clustering tool (accessed December 2020) placed strains 1139 and 1159 in clusters 401 and 420, respectively. Cluster 401 contained genomes of strains named as *R. erythropolis*, *R. enclensis* and *R. qingshengii*, including the type strains for *R. qingshengii* JCM 15477 (IMG/M genome ID 2744054616) and djl-6-2. Pairwise ANI comparisons of selected genomes in this clique are detailed in Table S1. There were two available genome assemblies for JCM 15477: they showed >99% ANI similarity but < 36% AF similarity, whereas other *R. qingshengii* genomes showed >98% ANI and >89 AF similarity, indicating detectable differences between the archived genomes of JCM 15477. Cluster 420 contains genomes for strains named as *R. erythropolis*, including the NCBI strain used as the reference genome for this species and type strain, CCM 2595, 1159 (which is annotated as *R. erythropolis* in IMG/M and NCBI databases) plus species named as *R. opacus*, *R. rhodochrous* or unclassified species. Pairwise ANI analysis between genomes in the two ‘*erythropolis*’ clusters suggests that the taxonomy of several strains currently named as *R. enclensis*, *R. rhodochrous*, *R. opacus* and *R. baikonurensis* (type strain JCM 18801) requires reconsideration, as ANI data indicate that these strains are all the same species.

### 3.6. Taxonomy of Williamsia Strains Based on Mycolic Acid and Genome Sequence Analyses

Bound and unbound mycolic acids were analysed from stationary phase cultures of strains 1135 and 1138 by UPLC–MS/MS (Table S2). Major MA components (>20% relative abundance) in strain 1135 contained 52–58 carbons with three double bonds and 54–58 carbons with 2 to 4 double bonds in strain 1138. MA detected under the growth conditions used for the latter stain also included components with 60 carbons and up to five double bonds in the bound MA fraction, while prior analysis of MA separated by thin layer chromatography followed by flame ionization–thermal conductivity/MS/MS showed minor components with smaller carbon chains (31–44 carbons, 0 to 5 double bonds) [41]. Multiple isomers of all MA major components were detected (Table S2).

Hierarchical clustering based on pfam functions for all *Williamsia* species with genomes archived in the IMG/M database (accessed May 2021) (Figure S3) showed a distinct lineage for strain 1135 while strain 1138 clustered with *W. limnetica* type strain, DSM 45521. Multiple sequence alignment of 16S rRNA genes obtained from archived genome sequences for the three strains showed >98.9% alignment scores, but also indicated small signature differences between these strains (Figure S4). Pairwise ANI comparisons showed 87.4 and 87.6% similarity between 1135 and 1138 to DSM 45521, respectively, and 75.7 and 79.5% AF similarity to DSM 45521, respectively. Strain 1135 showed 91.1% ANI and 78.9% AF similarity to 1138, affirming that these *Williamsia* strains are distinct from their nearest neighbour, DSM 45571, and from each other. 

### 3.7. Biochemical Characterization of the Rhodococcus Strains 1139 and 1159

Given that *Rhodococcus* strains 1139 and 1159 clustered with mesophilic *Rhodococcus* strains which included species described as heterotypic synonyms, further biochemical analyses were undertaken to determine whether these strains displayed differentiating traits, considering their origin on Macquarie Island (with oceanic geology, exposure to sea-spray and sub-Antarctic temperatures [42]).

#### 3.7.1. Enzymatic Characterization of Bacterial Strains and Carbon Source Utilization

The API ZYM test showed *Rhodococcus* sp. 1139 and 1159 were both strongly positive for leucine arylamidase, alkaline phosphatase, acid phosphatase, esterase lipase (C8), α-glucosidase and β-glucosidase, and showed weaker but detectable activity for esterase (C4), lipase, valine acrylamidase, cystine arylamidase, α-chymotrypsin and napthanol-AS-Bl-phosphohydrolase (Table S4). Examination of the genomes detected all genes in the ureide pathway for breakdown of nucleosides, including urease, although this was not experimentally tested here. Both strains showed similar µ_max_ on MSM-F and grew on glycerol. Growth rate on *D*-glucose in MSM was low but supplementing MSM–glucose with yeast extract increased this 3–4-fold for both strains. However, the highest µ_max_ was found on *D*-sorbitol and *D*-fructose for both strains (Figure S5).

#### 3.7.2. Identifying Preferred Nitrogen Sources and Impact of Phosphate Ion Concentration

*Rhodococcus* sp. 1139 and 1159 were initially screened in MSM-F media with the normally-used nitrogen source, NH_4_H_2_PO_4_ (AP), replaced with different potential nitrogen sources (urea and sulphate counter-ion with ammonium and nitrate), and combinations of these (Figure S6). Both strains could utilize ammonium, nitrate and urea as nitrogen sources to varying degrees.

When the phosphate ion concentration was adjusted to account for the decrease in total phosphate when NH_4_H_2_PO_4_ was replaced (phosphate-adjusted MSM-F media, -P), providing 25% more phosphate ion, both strains showed significant differences in µ_max_ and maximum final OD_600_ for all the nitrogen sources. Growth on ammonium sulphate (AS) was higher than AP when both were supplemented at 2 g/L, reflecting the higher concentration of nitrogen in AS; the higher concentration of phosphate in AS-P further improved growth.

#### 3.7.3. Salt Tolerance

*Rhodococcus* sp. 1139 and 1159 were cultured in MSM-F broths supplemented with 1 to 15% NaCl. Both *Rhodococcus* strains, 1139 and 1159, could tolerate up to 7% NaCl.

#### 3.7.4. Strains 1139 and 1159 Are Psychrotolerant and Predicted to Survive at Sub-Zero Temperatures

Bacterial strains were cultured in MSM-F at temperatures between 0 to 45 °C to determine the temperature for fastest growth rate (T_opt_) (Figure S7a). The experimentally-determined temperature range for growth was 0 to 35 °C with fastest growth rate at 30 °C for both 1139 and 1159. It was determined from the nonlinear model that these strains were predicted to survive at very low temperatures; the T_min_ was calculated as −9.5 ± 1.4 and −12 ± 0.8 °C and T_max_ as 35.3 ± 2.5 and 37.5 ± 0.5 °C, respectively, which is in good agreement with the measured growth ranges (Table S5). One example of the nonlinear fitted model is shown in Figure S7b.

#### 3.7.5. MA Analysis by UPLC–MS/MS

Table S6 summarizes the ESI–MS profiles for the major bound and unbound MA detected in the sub-Antarctic *Rhodococcus* strains. The MA found in *Rhodococcus* strains 1139 and 1159 had a wide range of carbon chain lengths, ranging from 34 to 58 with up to five double bonds. The major bound MA components had 36 to 46 carbons and one or two double bonds and tandem quadrupole MS data revealed 2–4 isomers for the major molecular species. The α-branch of these *Rhodococcus* strains ranged from 10 to 19 carbons with 0 to 1 double bonds and the meromycolate branch ranged from 24 to 44 carbons with 0 to 5 double bonds. The major bound MA in *Rhodococcus* spp. 1163 and 1168 contained 44–48 carbons, mainly with one or two double bonds, and longer-chained MA (up to 54 carbons) were detected as minor (relative abundance 20–62%) components, differentiating these novel species from 1139 and 1159. Strain 1163 also had a higher proportion of larger MA (50:2, 58:4) in the unbound fraction.

#### 3.7.6. TAG Analysis by UPLC–MS/MS

TAG molecular species eluted from the UPLC column between 6.6 and 9.5 min. TAGs were partially resolved into broad peaks consisting of several individual TAG molecular species. Mass spectra of these regions were averaged to determine the types of TAG present as [M + NH_4_]^+^ and [M + Na]^+^ adduct ions (Table S7). ESI–MS profiles of carbon units in TAG of *Rhodococcus* strains ranged from 46 to 52 with 1 to 3 double bonds.

#### 3.7.7. FAME Analysis by GC–MS

The carbon chain length of fatty acids (FA) ranged from 12 to 22 with up to one double bond in both *Rhodococcus* strains (Table S8). The major FA detected were C_16:0_ (31.2%), C_18:1_ (20.2%) and 10-methyl C_18:0_ (21.2%). Other minor FAs detected included C_16:1_ (two isomers, 10.4%), C_14:0_ (5.3%), C_18:0_ (2.2%) and very minor relative proportions (<2%) of longer-chained (C_19:1_–C_22:1_) and branched-chained FA.

## 4. Discussion

Genome-based taxonomic classification of bacteria is being applied increasingly, in parallel with developing standards for defining the lineages in several phyla and genera, including the Actinobacteria [25,43,44]. Sequence quality depends on several parameters, including the suitability of the DNA provided to meet criteria for use in next-generation sequencing, particularly providing templates for long reads for high-fidelity genome assembly and determining GC content [45]; selecting appropriate methodologies for DNA preparation is a crucial factor in phylogenomics. There are several commercial gDNA extraction kits available which have been successfully used for genome sequencing of Actinobacteria and mycolate-containing *Corynebacteriales* strains, as demonstrated by the increasing number of *Rhodococcus* and other genomes archived in the NCBI Genome Assembly database. Genome assembly quality underpins nomenclature and taxonomy when identifying species clusters through ANI comparisons with type strains, as employed as one of the tools in the Genome Taxonomy Database (GTDB) [46]. This is exemplified by reviewing the current number of *Rhodococcus* assemblies examined in the GTDB, where 20 of 384 assemblies (accessed December 2020) failed the quality check using CheckM [47], based on completeness, contamination or strain heterogeneity. Consequently, these 20 examples of both named and unclassified *Rhodococcus* spp. could not be assigned to any species cluster. It was also noted here that the two archived genome sequences for the type strain of *R. qingshengii* JCM 15477 showed different AF values, which may indicate that caution is required when selecting a ‘type strain’ genome assembly for comparative taxonomic purposes.

We were able to shotgun sequence six unclassified sub-Antarctic *Corynebacteriales* strains [27,28,29] after optimizing gDNA extraction using a method based on those described by Marmur [14,15]. This method was only successful when the strains were cultured in broths containing concentrations of glycine that were highly growth-inhibitory, which predisposed cells to lysozyme and proteinase K digestion. We assessed the quality of the gDNA using fragment size and size distribution data (Fragment Analyser, GQN) plus protein/DNA and RNA/DNA ratios, in addition to data provided by Macrogen Inc during sequencing (Q30, phred quality score) and after assembly using ABySS software (N50, L50, number of contigs and GC content) (see Table 2). The assemblies for all six strains passed CheckM in the GTDB and were assigned to species clusters, affirming the quality of the gDNA used to explore their taxonomic position and the phylogeny described herein.

Some bacteria are highly or moderately sensitive to growth inhibition by glycine, while others are resistant; there is no conclusive relationship between the sensitivity and bacterial physiological properties such as their Gram staining status, morphology or aerobic and anaerobic nature [48]. Sensitivity to growth inhibition also varies among bacterial species and strains, not with genus [20]. In this study, all the *Corynebacteriales* strains were moderately resistant to lower concentrations of glycine. However, *Williamsia* sp. 1135 was more sensitive to higher concentrations of glycine than *Williamsia* sp. 1138. Similar observations were previously reported for *Corynebacterium* species [19], where *C. mediolanum* was completely inhibited by 1.25% glycine, whereas *C. insidiosum* was similarly inhibited by 0.8% glycine. This demonstrates the need to evaluate each strain for the inhibitory effects of glycine prior to cultivation for DNA extraction if difficulties arise in using kits or other wet-lab procedures for cell lysis.

The results show that higher concentrations of glycine (4.0–4.5%) in the culture media facilitated enzymatic cell disruption of the sub-Antarctic strains, increasing the DNA yield for both the classical extraction method and commercial kit methods. A longer incubation period was required to disrupt cells grown in 0 and 2% MSM-G and the strains showed variability in the incubation time required to achieve visible lysis (notably the novel species of *Rhodococcus*, 1163 and 1168 required longer enzymatic treatment than the other *Rhodococcus* species, even after growth in 4.5% glycine). The need to optimize enzyme treatment periods was noted previously for kit-based extraction of DNA from actinobacterial cells not cultured in glycine-enriched media [17,18,49]. *Williamsia* sp. 1138, selected for optimizing gDNA extraction methods, was particularly recalcitrant to lysis. The size range of MA molecules detected in both sub-Antarctic *Williamsia* strains was in line with publications defining the novel genus (50–56 carbons) [50] and *W. limnetica* (50–58 carbons) [51], the closest neighbour phylogenetically. Under the growth conditions used, *Williamsia* sp. 1138 showed a higher size range of MA than the rhodococci, up to 60 carbons and multiple double bonds, which may contribute to the difficulty in cell lysis during gDNA extraction. *Williamsia* species are more rarely detected in environmental samples assessed following community DNA extraction, despite occupying similar ecological niches to *Rhodococcus* species; greater difficulty in cell lysis may contribute to these observations. The cell surface structures of *Rhodococcus* species vary within the genus, with some displaying relatively shorter MA (28–46 carbons) and others with longer chains (34–54 carbons) [52], in addition to a range of complex covalently-bound and unbound lipid plus carbohydrate structures [53]. The novel *Rhodococcus* spp., 1163 and 1168, contained a higher proportion of longer-chained MA (50–54 carbons) (Table S6) which may also have contributed to their resistance to lysis by enzymatic treatments, again emphasizing the need for optimizing gDNA extraction protocols for phylogenomic purposes when handling new species.

Although 16S rRNA gene sequencing is commonly used to determine phylogenomic relationships between bacterial strains, it is considered a poor predictor of proteome similarity [40] and was unable to resolve some genera and species in the phylum Actinobacteria [25]. While other genes, such as *rpoB* or groups of genes used in multi-locus sequence typing, may be better predictors, genome-wide measures of similarity, ANI and AF, strongly correlated with proteome similarity in *Streptomyces* and *Bacillus* without limiting analyses to the core genomes to eliminate input from genes recently acquired from horizontal transfer [40]. Neighbour-joining phylogenetic trees based on 16S rRNA genes, and *alkB* for strains 1163 and 1168, plus ANI-based whole-genome sequence cluster analyses, clearly indicated that the two *Williamsia* strains 1135 and 1138 and the *Rhodococcus* strains 1163 and 1168 are likely novel species, as they formed distinct clades. In contrast, *Rhodococcus* strains 1139 was phylogenetically close to *R. qingshengii* and 1159 to *R. erythropolis*, based on 16S rRNA gene sequences, ANI analyses and phylogenetic trees based on pfam or COG clustering. However, *Rhodococcus* sp. 1139 was also phylogenetically and biochemically closely related to *R. degradans* (see Table S9, which summarizes the biochemical and genetic traits of strains 1139 and 1159 together with published data for type strains of *R. degradans*, *R. qingshengii* and *R. erythropolis*). Following the original description of *R. erythropolis* in 1977 [54] as a member of the genus *Rhodococcus* (formerly *Nocardia*), it has become increasingly recognized that several validly published species of *Rhodococcus* are closely related to *R. erythropolis* and are members of the ‘*erythropolis/qingshengii/enclensis*’ group. The original description of *R. degradans* (CCM 4446 = HA1) as a distinct species [23] was based on several standard parameters, including 16S rRNA, *gyrA* and *catA* sequence comparisons, ribotyping, protein fingerprinting and DNA–DNA hybridisation. The authors noted that this proposed new species was phylogenetically related to *R. globerulus*, *R.* *baikonurensis*, *R. qingshengii* and *R. erythropolis* (the ‘16S rRNA gene clade’) but was differentiated based on carbon source usage. However, it was also noted that there were several disparities between carbohydrate utilisation tests when using kits and test-tube cultures. Our data for growth on different carbon and nitrogen sources was based on measuring the maximum growth rates and extent of growth, providing a stronger experimental platform for determining carbon source usage. Furthermore, the basal medium formulation (counter-ions for nitrogen sources, level of phosphate and presence of yeast extract in the MSM) impacted on the growth rates on some carbon sources, suggesting that strain differentiation based on carbon source utilization patterns is problematic given that outcomes depend on methodology and media used. These observations also indicated that any subsequent media optimization for biomass yield and TAG synthesis would need to evaluate the ratio of nitrogen, phosphate and sulphate ion concentrations in addition to the ratio of carbohydrate to nitrogen, which is considered a critical factor in TAG synthesis [7]. Table S9 shows that there are few biochemical traits which can differentiate between *R. erythropolis*, *R. qingshengii* and *R. degradan*, and between the type strains of these species and sub-Antarctic strains 1139 and 1159. The possible exceptions are higher salt tolerance and lowest minimum growth temperature, although it is difficult to be conclusive given that our data was based on longer incubation periods (up to 4 months for growth temperature experiments) relative to the methods used when describing the relevant type cultures. The genome for strain DSM 6344 (described as *R. erythropolis* = HA1 in the German culture collection and *R. degradans* for the equivalent strain in the Belgium collection, LMG 28633 = HA1) is now available (GenBank assembly accession GCA_009863335.1) [24]. Lee et al. [24] noted that *R. degradans* is a later heterotypic synonym of *R. qingshengii* [55], adding to the prior publications indicating that *R. jialingiae* [26] and *R. enclensis* [25] are also later heterotypic synonyms of *R. qingshengii*. Using a species delineation ANI point of 98%, and >60% AF, proved useful in classifying the sub-Antarctic *Rhodococcus* species, which is higher than the typically used point of 95% (see [45]); using 95% would place all of these named species into one. While further taxonomic tools will likely emerge to assess phylogenetic relationships between closely related bacteria, rationalisation of the nomenclature within the ‘*erythropolis*’ group may provide greater clarity in two areas: establishing claims of novel biotechnological prowess of new isolates; and tracking fundamental research on the biochemical and regulation systems in this group.

## 5. Conclusions

Prior culture in media supplemented with growth-inhibitory concentrations of glycine improved cell lysis for six lysozyme-resistant sub-Antarctic *Rhodococcus* and *Williamsia* isolates, using a classical method for gDNA extraction. Two extraction kits also yielded gDNA with fragment sizes suitable for third-generation sequencing. However, one kit only produced high-quality yields of DNA from *E. coli*, while the fragment size distribution for gDNA from the sub-Antarctic strains failed quality evaluation for genomic sequencing, although it was adequate for PCR amplification of 16S rRNA genes. Phylogenetic analysis of the sequenced genomes indicated that four sub-Antarctic strains are novel species which contained longer-chained mycolic acids, which may have contributed to their resistance to lysis. Two strains were classified as known species of *Rhodococcus*, which displayed biochemical traits expected of *R. qingshengii* (1139) and *R. erythropolis* (1159) and were both psychrotrophic. ANI clustering and further pairwise analyses indicated that delineation points of ANI similarity >96.5% and AF >60% were useful in classifying strains 1139 and 1159. However, ANI similarity of >98% was required to differentiate between strains in two ANI genome clusters which included *R. erythropolis* and *R. qingshengii* type strains. Several strains in these two clusters are currently named as *R. enclensis, R. baikonurensis, R. opacus* and other *Rhodococcus* species, indicating that the taxonomic position of these strains in the *R. erythropolis* group requires clarification.

## Figures and Tables

**Table 1:** Average scores for DNA quality and quantity following extraction from *Williamsia* sp. 1138 cells cultured in MSM supplemented with 0, 2 and 4.5% glycine.

Parameters	Method 1 Isolate II Genomic DNA Kit (Bioline)				Method 2 Ultraclean Microbial DNA Isolation Kit (MO BIO)				Method 3 QIAAMP Mini Kit (Qiagen)				Method 4 Classical Extraction		
	0% ^a^	2% ^a^	4.5% ^a^	*E. coli*	0% ^a^	2% ^a^	4.5% ^a^	*E. coli*	0% ^a^	2% ^a^	4.5% ^a^	*E. coli*	0% ^a^	2% ^a^	4.5% ^a^
**Mean concentration of DNA (ng/µL) ± s.d. ^b^**	10 ± 1	39 ± 3.2	69 ± 2	295 ± 2	25.7 ± 1.1	45.7 ± 4.9	60.2 ± 6.7	74 ± 3.5	17 ± 2.6	42.2 ± 3.2	71.7 ± 3.5	79.4 ± 2.5	1.5 ± 0.35	6 ± 1.64	30.4 ± 4.1
**Mean GQN ± s.d. ^b^**	0.9 ± 0.07	1.0 ± 0.3	0.2 ± 0.4	6.5 ± 0.3	5.9 ± 0.06	8.1 ± 0.04	8.4 ± 0.12	9.2 ± 0.03	6.5 ± 0.56	7.8 ± 0.35	7.6 ± 0.35	8.6 ± 0.1	1.8 ± 0.04	6.5 ± 0.28	9.0 ± 0.07
**Fragment size range (bp) ^b^**	823–9763	557–1321	27–388	991–39,079	2019–40,109	2849–54,726	2755–54,903	1913–48,525	8549–49,782	7605–55,301	2891–50,707	6787–52,428	0–14	22,636–44,933	16,591–42,748
**Average fragment size (bp) ^b^**	9554	490	128	13,975	21,246	18,923	17,284	26,027	24,249	26,555	23,410	32,406	2.0	33,581	28,430
**Mean A_260_/A_280_** **± s.d. ^c^**	2.0 ± 0.09	2.0 ± 0.04	2.0 ± 0.06	2.1 ± 0.03	2.0 ± 0.02	1.99 ± 0.04	1.88 ± 0	1.98 ± 0.03	2.1 ± 10.04	2.1 ± 0.01	2.1 ± 0.01	2.1 ± 0.09	1.96 ± 0.09	1.69 ± 0.04	1.70 ± 0.06
**Mean A_260_/A_230_** **± s.d. ^c^**	2.1 ± 0.03	1.9 ± 0.12	1.8 ± 0.03	1.9 ± 0.06	1.3 ± 0.12	1.4 ± 0.16	1.6 ± 0.06	1.6 ± 0.09	1.1 ± 0.03	2.1 ± 0.03	2.1 ± 0.03	2.1 ± 0.06	0.91 ± 0.3	1.65 ± 0.12	1.71 ± 0.03
**Final volume (µL)**	50	50	50	50	50	50	50	50	100	100	100	200	50	50	50

^a^ 0, 2, 4.5% are the concentrations of glycine in the culture medium for sub-Antarctic strains; ^b^ Concentration of DNA and GQN number obtained from Fragment Analyser analyses; ^c^ A_260_/A_280_ and A_260_/A_230_ ratio were obtained from NanoDrop spectrophotometry; s.d. = standard deviation.

**Table 2:** Quality and quantity scores for gDNA obtained from large-scale (50 mL) cultures of six sub-Antarctic strains.

Strain Name	ng/µL ^a^	GQN ^a^	Fragment Size Range (Kbp) ^a^	Average Size (Kbp) ^a^	A 260/280 ^b^	A 260/230 ^b^	Q30 (%) ^c^	N50 ^d^ (Kbp)	L50 ^d^	Contigs ^d^	GC Content (%) ^d^
*Williamsia* sp. 1135	66.1	8.1	13.28–44.50	28.721	1.92	1.84	64.36	142.77	14	109	64.7
*Williamsia* sp. 1138	319	9.8	57.24–62.78	59.424	1.93	1.93	63.92	191.09	12	54	64.8
*R. qingshengii* 1139	169.7	9.3	41.57–56.44	48.133	1.94	1.75	62.70	94.97	24	192	62.3
*R. erythropolis* 1159	106.2	8.7	11.63–48.44	27.142	1.78	1.78	65.11	196.55	13	114	62.3
*Rhodococcus* sp. 1163	40.5	8.7	15.73–47.69	32.799	1.99	1.87	64.67	329.55	5	43	62.3
*Rhodococcus* sp. 1168	66.3	9.5	9.72–51.78	27.334	2.01	1.88	66.25	154.59	10	97	62.1

^a^ DNA concentration and GQN obtained from Fragment Analyser data. ^b^ A_260_/A_280_ and A_260_/A_230_ ratio were obtained by Nano Drop spectrophotometry. ^c^ Obtained from Macrogen: Q30 is the % of reads that have a phred quality score of over 30. ^d^ Obtained after assembly using ABySS software; N50 is the length of the shortest contig that provides 50% of the genome when information in contigs above this size is summed [1]; DNA GC content of assembled sequences.

**Table 3:** ANI and AF comparisons for sub-Antarctic strains with type strains of *R. erythropolis* and *R. qingshengii* (panel A) and the closest neighbours of strains 1163 and 1168 (panel B), highlighting species identity between strains of *R. erythropolis* (orange shading) or *R. qingshengii* (green shading).

**Panel A**									
**Strain No.**	**Species**	**Provenance**	**Pairwise ANI and AF (%) ^a^**						
			**CCM 2595**	**NBRC** **15567**	**TUHH-12**	**JCM 15477**	**Djl-6-2**	**1139**	**1159**
CCM 2595	*R. erythropolis*	NCBI reference genome for the species		98.83 AF = 88.64	95.55 AF = 64.79	95.60 AF = 79.47	95.57 AF = 85.27	95.67 AF = 81.41	98.86 AF = 84.01
NBRC 15567 = JCM 3201 ^b^	*R. erythropolis*	Type strains, https://lpsn.dsmz.de	98.83 AF = 99.99		95.49 AF = 65.56	95.49 AF = 81.12	95.54 AF = 85.42	95.53 AF = 82.11	98.78 AF = 84.47
TUHH-12	*R. qingshengii*	NCBI-cited genome publication for this species (2015)	95.547 AF = 72.99	95.49 AF = 71.14		98.58 AF = 68.04	98.77 AF = 72.62	98.73 AF = 69.10	95.43 AF = 68.31
JCM 15477 ^c^	*R. qingshengii*	Type strain, https://lpsn.dsmz.de	95.60 AF = 89.60	95.49 AF = 88.16	98.57 AF = 68.11		98.83 AF = 89.45	98.67 AF = 85.23	95.47 AF = 83.86
Djl-6-2	*R. qingshengii*	Related to original isolate djl-6 type strain, https://lpsn.dsmz.de	95.57 AF = 85.47	95.54 AF = 85.32	98.79 AF = 66.68	98.82 AF = 82.23		98.91 AF = 82.90	95.53 AF = 80.41
1139	*Rhodococcus* sp.	This study	95.66 AF = 89.01	95.52 AF = 86.46	98.73 AF = 67.09	98.67 AF = 82.59	98.91 AF = 87.35		95.56 AF = 83.34
1159	*Rhodococcus* sp.	This study	98.86 AF = 92.59	98.78 AF = 89.77	95.44 AF = 66.72	95.48 AF = 81.83	95.54 AF = 85.57	95.55 AF = 83.95	
**Panel B**									
**Strain**	**Species**	**Provenance**	**Pairwise ANI and AF (%)**						
			PAMC 28707			1163		1168	
PAMC 28705 PAMC 28707	*Rhodococcus* sp. (identical strains)	Published genome Korean Polar Research Institute (IMG/M)				94.50 AF = 84.60		93.58 AF = 74.36	
1163	*Rhodococcus* sp.	This study	94.50 AF = 80.13					93.75 AF = 74.20	
1168	*Rhodococcus* sp.	This study	93.55 AF = 79.27			93.74 AF = 83.80			

^a^ Average nucleotide identity (ANI) and alignment fraction (AF) were calculated using the IMG/M pairwise ANI tool; shading in panel A shows identity between species. ^b^ Genome BLAST (NCBI) indicated that these two strains are identical. ^c^ There are two genome sequences in IMG/M for the type strain JCM 15477: genome ID 2744054616 (NCBI GCF_001646745.1) was used for ANI pairwise comparisons here; ANI analysis of JCM 15477 genome ID 2734481946 (NCBI GCA_001313445.1) showed >99% ANI similarity but < 35% AF with genome ID 2744054616 and other strains named as *R. qingshengii* (Table S1).

## Supplementary Materials

The following are available online at https://www.mdpi.com/article/10.3390/microorganisms9061253/s1. Figure S1: Hierarchical clustering based on pfam functions of all *Rhodococcus* species with genomes archived in the IMG/M database (accessed March, 2020); Figure S2: Phylogenetic tree for all unclassified species of *Rhodococcus* (constructed using NCBI Genome BLAST, Taxonomy Browser); Figure S3: Hierarchical clustering based on pfam functions of all *Williamsia* species with genomes archived in the IMG/M database (accessed May, 2021); Figure S4: Multiple sequence alignment (Clustal 2.1) of 16S rRNA genes from the genome sequences of *Williamsia* sp. 1135 and 1138 and *Williamsia limnetica* (DSM 45521^T^) archived in the IMG/M database (accessed February 2021); Figure S5: Maximum growth rates (µ_max_, h^−1^) and final OD_600_ on different carbon sources of bacterial strains: (a) *Rhodococcus* sp. 1139 and (b) *Rhodococcus* sp. 1159; Figure S6: Maximum growth rates (µ_max_, h^−1^) and final OD_600_ on different nitrogen sources of bacterial strains: (a) *Rhodococcus* sp. 1139 and (b) *Rhodococcus* sp. 1159; Figure S7: Impact of temperature on growth kinetics of *Rhodococcus* strain 1139 and 1159. (a) Maximum growth rates (µ_max_, h^−1^) at different temperatures during culture in MSM-F broth; (b) one example of the nonlinear fitted model for strain 1159; Table S1: ANI and AF pairwise comparison of sub-Antarctic *Rhodococcus* isolates with other *Rhodococcus* strains selected from ANI genome clusters containing species in the *R. erythropolis* group (‘*erythropolis/qingshengii/enclensis*’) available at the IMG/M site (accessed in March 2020); Table S2: Mycolic acids (relative abundance ≥ 20%) of *Williamsia* spp. 1135 and 1138; Table S3: ANI and AF pairwise comparison of sub-Antarctic *Williamsia* isolates 1135 and 1138 with other *Williamsia* species for genomes available at the IMG/M site (accessed in May 2021); Table S4: Enzymatic characteristics of *Rhodococcus* strains 1139 and 1159; Table S5: Experimentally determined and modelled temperature profiles of *Rhodococcus* strains 1139 and 1159; Table S6: Mycolic acids (relative abundance ≥ 20%) of sub-Antarctic *Rhodococcus* strains; Table S7: Major triacylglycerols found in *Rhodococcus* strains 1139 and 1159; Table S8: Total fatty acids composition of *Rhodococcus* strains 1139 and 1159; Table S9: Phenotypic and genotypic characteristics of *Rhodococcus* strains 1139 and 1159 and closely related type strains.
