# Supplementary material for: Benchmarking DNA Extraction Methods for Phylogenomic Analysis of Sub-Antarctic Rhodococcus and Williamsia Species
Source: Microorganisms. 2021 Jun 9;9(6):1253. doi: 10.3390/microorganisms9061253 (PMC8227252; doi:10.3390/microorganisms9061253)
Supplement: Supplementary file 1 [file microorganisms-09-01253-s001.zip › microorganisms-1210990-supplementary.pdf]

Supplementary Materials

Benchmarking DNA extraction methods for phylogenomic analysis of sub-Antarctic *Rhodococcus* and *Williamsia* species

Akhikun Nahar [1,*], Anthony L. Baker [1], David S. Nichols [2], John P. Bowman [1] and Margaret L. Britz [1,*]

[1] Tasmanian Institute of Agriculture, University of Tasmania, Hobart, Tasmania, Australia, 7005; Akhikun.nahar@utas.edu.au; Anthony.baker@outlook.com.au; john.bowman@utas.edu.au; Margaret.britz@utas.edu.au

[2] Central Science Laboratory, Division of Research, University of Tasmania, Hobart, TAS 7005, Australia; david.nichols@utas.edu.au; david.nichols@utas.edu.au

* Correspondence: Akhikun.nahar@utas.edu.au (A.H.); Margaret.britz@utas.edu.au (M.B.)

Figure S1. Hierarchical clustering of all *Rhodococcus* species with genomes archived in the IMG/M database based on pfam functions. Accessed March, 2020.
Figure S2. Phylogenetic tree for all unclassified species of *Rhodococcus* (constructed using NCBI Genome BLAST, Taxonomy Browser)
Figure S3. Maximum growth rates (μ_{max}, h^{-1}) and final OD_{600} on different carbon sources of bacterial strains: a) *Rhodococcus* sp. 1139 and b) *Rhodococcus* sp. 1159. MSM=Minimal salt media without any carbon source; MSM-G= Minimal salt media with D-glucose; YE= Yeast extract.
Figure S4. Maximum growth rates (μ_{max}, h^{-1}) and final OD_{600} on different nitrogen sources of bacterial strains: a) *Rhodococcus* sp. 1139 and b) *Rhodococcus* sp. 1159. P = phosphate adjusted media; AP = ammonium phosphate; AS = ammonium sulphate; SN = sodium nitrate.
Figure S5. Impact of temperature on growth kinetics of bacterial strains: a) Maximum growth rates (μ_{max}, h^{-1}) at different temperatures during culture in MSM-F broth; b) one example of the non-linear fitted model for strain 1159.

Table S1. Quality and quantity scores for gDNA obtained from large-scale (50 mL) cultures of six sub-Antarctic strains
Table S2. Enzymatic characteristics of six bacterial strains
Table S3. Experimentally determined and modelled temperature profiles of six sub-Antarctic strains
Table S4. Mycolic acid (relative abundance ≥20%) compositions of the two *Rhodococcus* bacterial strains
Table S5. Major triacylglycerols found in the two *Rhodococcus* bacterial strains
Table S6. Total fatty acids composition of the two *Rhodococcus* bacterial strains
Table S7. ANI and AF pairwise comparison of sub-Antarctic *Rhodococcus* isolates (1139, 1159, 1163 and 1168) with other *Rhodococcus* strains selected from ANI genome clusters containing species in the *R. erythropolis* group ('*erythropolis*/*qingshengii*/*enclensis*') available at the IMG/M site (accessed in March 2020)

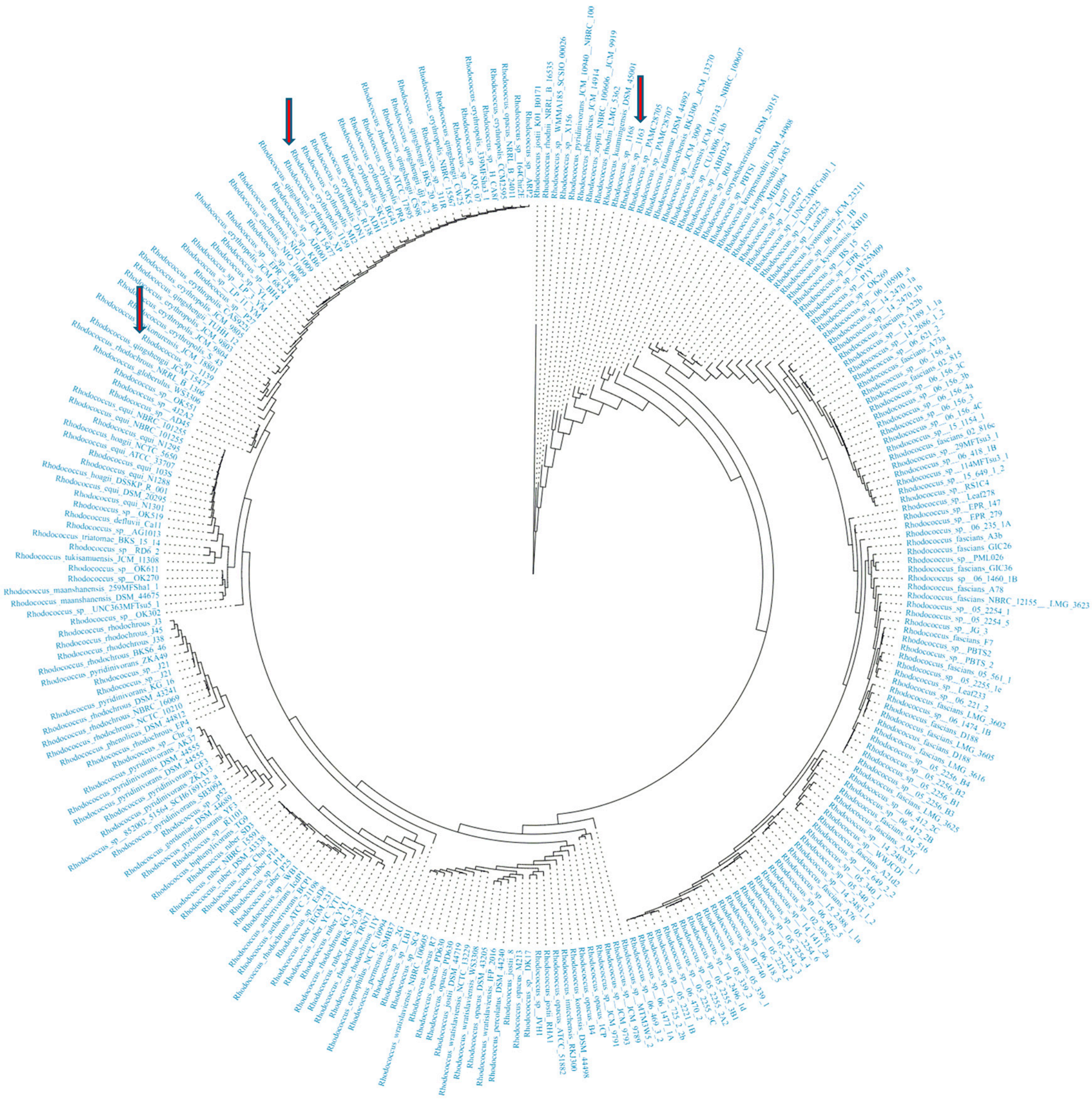

Figure S1. Hierarchical clustering of all *Rhodococcus* species with genomes archived in the IMG/M database based on pfam functions. Accessed March, 2020. Arrows point to the sub-Antarctic strains.

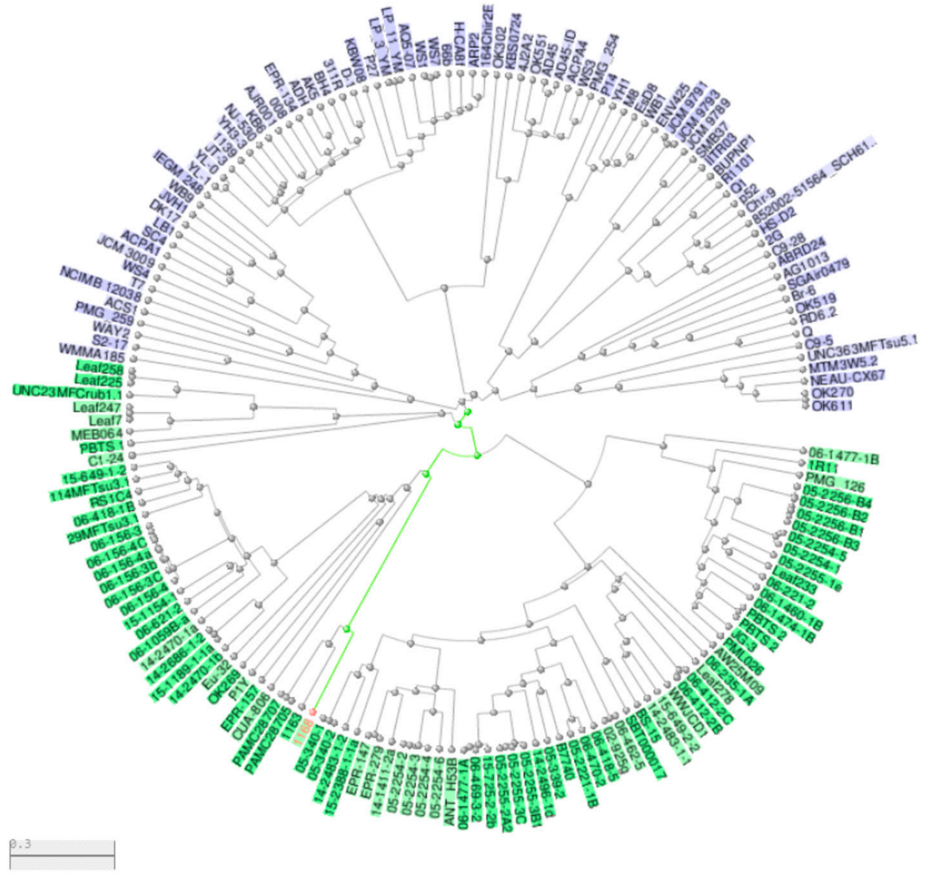

Figure S2. Phylogenetic tree for all unclassified species of *Rhodococcus* (constructed using NCBI Genome BLAST, Taxonomy Browser). Strain 1168 is in red.

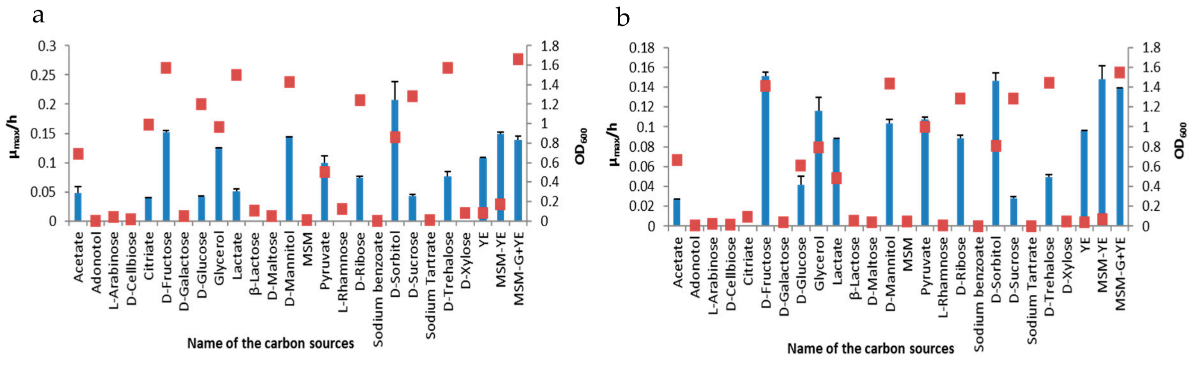

Figure S3. Maximum growth rates (μ_{max}, h^{-1}) and final OD_{600} on different carbon sources of bacterial strains: a) *Rhodococcus* sp. 1139 and b) *Rhodococcus* sp. 1159. MSM=Minimal salt media without any carbon source; MSM-G= Minimal salt media with *D*-glucose; YE= Yeast extract. Data are from a minimum of triplicates and standard deviations are shown for μ_{max}.

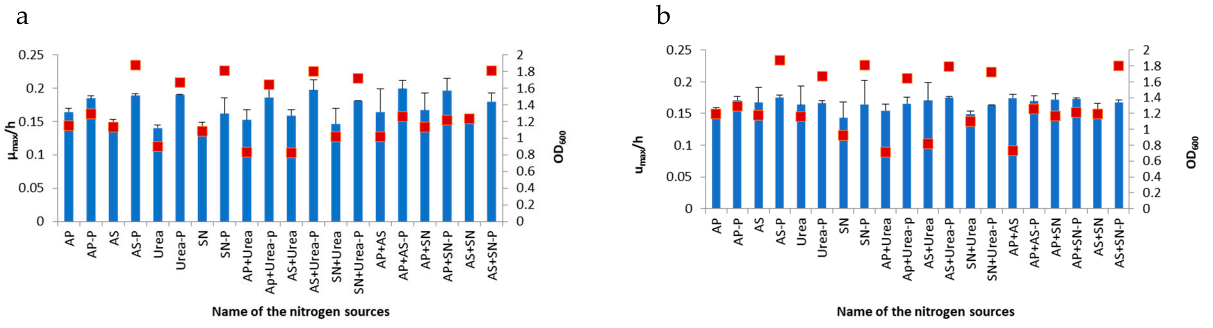

Figure S4. Maximum growth rates (μ_{max}, h^{-1}) and final OD_{600} on different nitrogen sources of bacterial strains: a) *Rhodococcus* sp. 1139 and b) *Rhodococcus* sp. 1159. P = phosphate adjusted media; AP = ammonium phosphate; AS = ammonium sulphate; SN = sodium nitrate. Data are from a minimum of triplicates and standard deviations are shown for μ_{max}.

The concentrations of nitrogen (N), phosphate (P) and sulphate (S) in the media were: AP, 17 mM N and 72 mM P; AP-P, 17 mM N and 89.3 mM P; AS, 30 mM N, 15 mM S and 54.7 mM P; AS-P, 30 mM N, 15 mM S and 72 mM P; Urea, 66.6 mM N and 54.7 mM P; Urea-P, 66.6 mM N and 72 mM P; SN, 24 mM N and 54.7 mM P; SN-P, 24 mM N and 72 mM P; AP+Urea, 41.8 mM N and 54.7 mM P; AP+Urea-P, 41.8 mM N and 80.8 mM P; AS+Urea, 48.3 mM N, 7.5 mM S and 54.7 mM P; AS+Urea-P, 48.3 mM N, 7.5 mM S and 72 mM P; SN+Urea, 53 mM N and 54.7 mM P; SN+Urea-P, 53 mM N and 72 mM P; AP+AS, 23.5 N and 63.2 mM P; AP+AS-P, 23.5 N and 80.8 mM P; AP+SN, 20.5 mM N and 63.2 mM P; AP+SN-P, 20.5 mM N and 80.8 mM P; AS+SN, 27 mM N, 7.5 mM S and 54.7 mM P; AS+SN-P, 27 mM N, 7.5 mM S and 72mM P. Note that the concentrations listed do not include trace amounts of sulfate from $FeSO_4$ and in the Instant Oceans salts mixture.

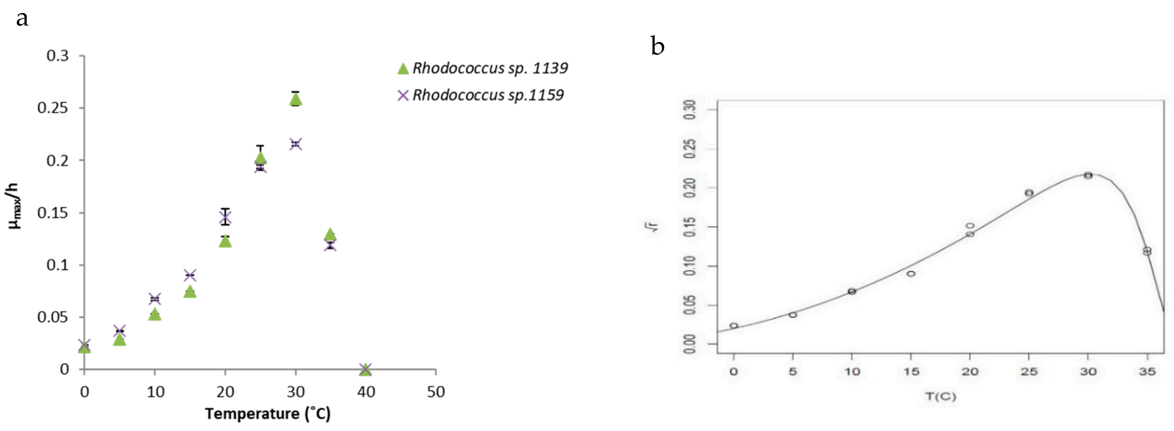

Figure S5. Impact of temperature on growth kinetics of bacterial strains: a) Maximum growth rates (μ_{max}, h^{-1}) at different temperatures during culture in MSM-F broth; b) one example of the non-linear fitted model for strain 1159.

Table S1. Quality and quantity scores for gDNA obtained from large-scale (50 mL) cultures of six sub-Antarctic strains

Strain name	Concentration (ng/µL)[a]	GQN[a]	Fragment size range (bp)[a]	Average size (bp)[a]	A 260/280[b]	A 260/230[b]	Q30 (%)[c]	N50[d]	L50[d]	Number of contigs[d]	GC content (%)[d]
Williamsia sp. 1135	66.1	8.1	13,283-44,499	28,721	1.92	1.84	64.36	142,767	14	109	64.7
Williamsia sp. 1138	319	9.8	57,242-62,779	59,424	1.93	1.93	63.92	191,087	12	54	64.8
R. qingshengii strain 1139	169.7	9.3	41,569-56,436	48,133	1.94	1.75	62.70	94,974	24	192	62.3
R. erythropolis strain 1159	106.2	8.7	11,629-48,443	27,142	1.78	1.78	65.11	196,546	13	114	62.3
Rhodococcus sp. 1163	40.5	8.7	15,726-47,685	32,799	1.99	1.87	64.67	329,545	5	43	62.3
Rhodococcus sp. 1168	66.3	9.5	9,721-51,781	27,334	2.01	1.88	66.25	154,590	10	97	62.1

[a] obtained from Fragment Analyzer analyses;

[b] A_{260}/A_{280} and A_{260}/A_{230} ratio were obtained from Nano Drop spectrophotometry;

[c] obtained from Macrogen: Q30 is the % of reads that have phred quality score of over 30;

[d] obtained after assembly using ABySS software; N50 is the length of the shortest contig that provides 50% of the genome when information in contigs above this size is summed [1]; DNA GC content of assembled sequences

Table S2. Enzymatic characteristics of six bacterial strains

Name of the enzymes[a]	*Rhodococcus* sp. 1139	*Rhodococcus* sp. 1159
Alkaline phosphatase	5	5
Esterase (C4)	1	1
Esterase lipase (C8)	3	4
Lipase	1	1
Leucine arylamidase	5	5
Valine arylamidase	2	2
Cystine arylamidase	±	1
Trypsin	0	0
α-chymotrypsin	1	±
Acid-phosphatase	5	5
Naphthanol-AS-Bl-phosphohydrolase	1	2
α-galactosidase	0	0
β-galactosidase	0	0
β-glucuronidase	0	0
α-glucosidase	5	5
β-glucosidase	4	4
N-acetyl-β-glucosaminidase	0	0
α-mannosidase	0	0
α-fucosidase	0	0

[a] API ZYM (bioMérieux, North Ryde, NSW, Australia) kits were used: 0 corresponds to a negative reaction and 5 corresponds to maximum intensity.

Table S3. Experimentally determined and modelled temperature profiles of six sub-Antarctic strains

Strains	T_{opt} (˚C)[a]	No growth (˚C)[b]	T_{min} (˚C)[c]	T_{max} (˚C)[c]
***Rhodococcus* sp. 1139**	30	40	-9.5±1.4	35.3±2.5
***Rhodococcus* sp. 1159**	30	40	-12±0.8	37.5±.5

[a] Highest μ_{max} determined from triplicate growth curves in MSM-F broth; [b] Temperatures where strains were unable to initiate growth in MSM-F broth; [c] Predicted minimum and maximum temperatures of growth from nonlinear fitted models of growth.

Table S4. Mycolic acid (relative abundance ≥20%) compositions of the two *Rhodococcus* bacterial strains

Name of the strains	[M-H]- (m/z) unbound mycolic acids	Number of Carbons: Number of unsaturated bonds	Relative intensity (%)	Mycolic acids (meromycolate/α-chain) with isomers	M-H]- (m/z) bound mycolic acids	Number of Carbons: Number of unsaturated bonds	Relative intensity (%)
Rhodococcus sp. 1139	605	40:1	90	28:1/12:0; 26:1/14:0; 24:1/16:0	521	34:1	45
	619	41:1	20	28:1/13:0; 27:1/14:0; 26:1/15:0, 25:0/16:1	549	36:1	92
	631	42:2	40	32:2/10:0; 30:2/12:0; 28:0/14:0; 26:1/16:1	687	46:2	100
	633	42:1	60	30:1/12:0; 28:1/14; 26:1/16	689	46:1	50
	647	43:1	20	31:1/12:0; 30:1/13:0; 26:1/17:0	701	47:2	20
	659	44:2	30	32:2/12:0; 30:2/14; 28:2/16	715	48:2	50
	661	44:1	35	32:1/12:0; 30:1/14; 28:1/16:0			
	699	47:3	20	31:2/16:1; 29:2/18:1			
	851	58:4	25	44:2/14:2; 43:4/15:0; 42:4/16:0; 39:4/19:0			
Rhodococcus sp. 1159	699	47:3	100	31:2/16:1; 29:2/18:1	521	34:1	31
	715	48:2	50	38:2/10:0; 36:2/12:0; 32:2/16:0	549	36:1	75
	717	48:1	22	32:1/16:0; 30:0/18:1	577	38:1	100
	835	57:5	30	43:3/14:2; 41:5/16:0; 39:4/18:1; 38:5/19:0	605	40:1	90
	851	58:4	32	43:4/15:0; 42:4/16:0; 39:4/19:0	633	42:1	68
					659	44:2	61
					687	46:2	48

Table S5. Major triacylglycerols found in the two *Rhodococcus* bacterial strains

Sample	*Rhodococcus* sp. 1139	*Rhodococcus* sp. 1159
46:1		+
48:1	+	+
48:2	+	+
49:1	+	
50:1		
50:2	+	+
50:3		+
51:1		+
51:2	+	
52:0		
52:1	+	+
52:2	+	+
52:3	+	

[a] + corresponds to the detection of triacylglycerols species

Table S6. Total fatty acids composition of the two *Rhodococcus* bacterial strains

**Fatty acids**	***Rhodococcus* sp. 1139**	***Rhodococcus* sp. 1159**
	Peak areas (%) of fatty acids[a]	
12:0	0.7	1.6
i14:0	-	-
14:0	5.3	6.1
14:1(n-7)	0.2	0.2
15:0	0.8	0.6
15:1*	-	-
16:0	31.2	34.6
16:1(n-9)	2.0	1.2
16:1(n-7)	8.4	5.6
i17:0	0.4	0.2
17:0	0.6	0.7
17:1(n-8)	0.7	0.1
18:0	2.2	9.3
18:1(n-9)	20.2	21.7
10Me18:0	21.2	9.4
19:1*	1.9	1.4
20:0	1.0	2.4
20:1*	0.2	0.9
21:1*	0.3	0.4
21:1*	0.3	0.4
21:2	-	-
22:0	2.3	2.9
22:1*	0.1	0.4

[a] - = < 0.1% of total fatty acid composition. Major fatty acids are highlighted; *isomers of fatty acids.

Table S7. ANI and AF pairwise comparison of sub-Antarctic *Rhodococcus* isolates (1139, 1159, 1163 and 1168) with other *Rhodococcus* strains selected from ANI genome clusters containing species in the *R. erythropolis* group (‘*erythropolis/qingshengii/enclensis*’) available at the IMG/M site (accessed in March 2020)

Genome1 ID	Genome1 Name	Genome2 ID	Genome2 Name	ANI1->2	ANI2->1	AF1->2	AF2->1
2561511168	Rhodococcus erythropolis CCM2595	2565956761	Rhodococcus qingshengii BKS 20-40	95.5834	95.5845	88.644	87.163
2561511168	Rhodococcus erythropolis CCM2595	2615840625	Rhodococcus enclensis NIO-1009	95.5575	95.5559	90.216	77.854
2561511168	Rhodococcus erythropolis CCM2595	2744054616	Rhodococcus qingshengii JCM 15477	95.5976	95.5959	89.597	79.467
2561511168	Rhodococcus erythropolis CCM2595	2816332270	Rhodococcus qingshengii CS98	95.6429	95.6423	88.648	84.696
2561511168	Rhodococcus erythropolis CCM2595	2816332322	Rhodococcus erythropolis 1159	98.8595	98.858	92.588	84.021
2561511168	Rhodococcus erythropolis CCM2595	2690315933	Rhodococcus qingshengii CW25	95.5969	95.5988	89.151	88.353
2561511168	Rhodococcus erythropolis CCM2595	2728369692	Rhodococcus baikonurensis JCM 18801	95.3512	95.3514	45.794	45.14
2561511168	Rhodococcus erythropolis CCM2595	2734481946	Rhodococcus qingshengii JCM 15477	95.0755	95.079	29.753	28.672
2561511168	Rhodococcus erythropolis CCM2595	2724679741	Rhodococcus sp. 1163	73.6562	73.6661	30.282	43.692
2561511168	Rhodococcus erythropolis CCM2595	2724679742	Rhodococcus sp. 1168	73.6667	73.6588	30.441	38.779
2615840625	Rhodococcus enclensis NIO-1009	2565956761	Rhodococcus qingshengii BKS 20-40	98.7424	98.7518	80.669	91.937
2615840625	Rhodococcus enclensis NIO-1009	2816332270	Rhodococcus qingshengii CS98	98.7297	98.7298	78.895	87.349
2615840625	Rhodococcus enclensis NIO-1009	2816332322	Rhodococcus erythropolis 1159	95.3579	95.3534	79.682	83.953
2615840625	Rhodococcus enclensis NIO-1009	2690315933	Rhodococcus qingshengii CW25	98.7524	98.7653	78.703	90.384
2615840625	Rhodococcus enclensis NIO-1009	2728369692	Rhodococcus baikonurensis JCM 18801	98.2311	98.2295	43.082	49.15
2615840625	Rhodococcus enclensis NIO-1009	2734481946	Rhodococcus qingshengii JCM 15477	97.9491	97.9477	27.653	30.889
2615840625	Rhodococcus enclensis NIO-1009	2724679741	Rhodococcus sp. 1163	73.624	73.6363	26.363	44.14
2615840625	Rhodococcus enclensis NIO-1009	2724679742	Rhodococcus sp. 1168	73.5923	73.6215	26.95	39.877
2617271037	Rhodococcus qingshengii TUHH-12	2561511168	Rhodococcus erythropolis CCM2595	95.5471	95.5472	64.793	72.99
2617271037	Rhodococcus qingshengii TUHH-12	2565956761	Rhodococcus qingshengii BKS 20-40	98.7648	98.7636	66.343	73.526
2617271037	Rhodococcus qingshengii TUHH-12	2615840625	Rhodococcus enclensis NIO-1009	98.7028	98.7027	70.923	68.994
2617271037	Rhodococcus qingshengii TUHH-12	2744054616	Rhodococcus qingshengii JCM 15477	98.5741	98.5739	68.111	68.037
2617271037	Rhodococcus qingshengii TUHH-12	2802429286	Rhodococcus enclensis NIO-1009	98.7028	98.7027	70.916	68.923
2617271037	Rhodococcus qingshengii TUHH-12	2816332270	Rhodococcus qingshengii CS98	98.7026	98.7028	65.521	70.504

2617271037	Rhodococcus qingshengii TUHH-12	2816332322	Rhodococcus erythropolis 1159	95.435	95.4277	66.717	68.305
2617271037	Rhodococcus qingshengii TUHH-12	2690315933	Rhodococcus qingshengii CW25	98.6899	98.6901	65.309	72.946
2617271037	Rhodococcus qingshengii TUHH-12	2728369692	Rhodococcus baikonurensis JCM 18801	98.4093	98.4096	43.879	48.726
2617271037	Rhodococcus qingshengii TUHH-12	2734481946	Rhodococcus qingshengii JCM 15477	97.8302	97.8279	29.603	32.16
2617271037	Rhodococcus qingshengii TUHH-12	2724679741	Rhodococcus sp. 1163	73.7044	73.7229	21.738	35.344
2617271037	Rhodococcus qingshengii TUHH-12	2724679742	Rhodococcus sp. 1168	73.6493	73.6852	22.405	32.414
2724679739	Rhodococcus sp. 1139	2561511168	Rhodococcus erythropolis CCM2595	95.6657	95.6564	81.408	89.012
2724679739	Rhodococcus sp. 1139	2565956761	Rhodococcus qingshengii BKS 20-40	98.8486	98.8617	83.113	89.319
2724679739	Rhodococcus sp. 1139	2615840625	Rhodococcus enclensis NIO-1009	98.7772	98.7713	86.845	81.736
2724679739	Rhodococcus sp. 1139	2617271037	Rhodococcus qingshengii TUHH-12	98.7292	98.7314	69.099	67.093
2724679739	Rhodococcus sp. 1139	2690315933	Rhodococcus qingshengii CW25	98.8275	98.8259	82.527	89.417
2724679739	Rhodococcus sp. 1139	2744054616	Rhodococcus qingshengii JCM 15477	98.6676	98.6659	85.228	82.585
2724679739	Rhodococcus sp. 1139	2802429286	Rhodococcus enclensis NIO-1009	98.7777	98.7718	87.143	81.717
2724679739	Rhodococcus sp. 1139	2816332270	Rhodococcus qingshengii CS98	98.765	98.7672	83.191	86.938
2724679739	Rhodococcus sp. 1139	2816332322	Rhodococcus erythropolis 1159	95.549	95.5641	83.95	83.335
2724679739	Rhodococcus sp. 1139	2816332450	Rhodococcus qingshengii djl-6-2	98.9102	98.9096	82.895	87.346
2724679739	Rhodococcus sp. 1139	2728369692	Rhodococcus baikonurensis JCM 18801	98.271	98.3886	43.928	46.904
2724679739	Rhodococcus sp. 1139	2734481946	Rhodococcus qingshengii JCM 15477	97.9077	97.9259	28.494	29.811
2724679739	Rhodococcus sp. 1139	2724679741	Rhodococcus sp. 1163	73.7193	73.7236	27.571	43.561
2724679739	Rhodococcus sp. 1139	2724679742	Rhodococcus sp. 1168	73.6348	73.634	28.258	39.28
2728369692	Rhodococcus baikonurensis JCM 18801	2802429286	Rhodococcus enclensis NIO-1009	98.2312	98.2328	49.21	43.095
2728369692	Rhodococcus baikonurensis JCM 18801	2615840625	Rhodococcus enclensis NIO-1009	98.2295	98.2311	49.15	43.082
2728369692	Rhodococcus baikonurensis JCM 18801	2816332322	Rhodococcus erythropolis 1159	95.2327	95.2236	47.315	43.715
2728369692	Rhodococcus baikonurensis JCM 18801	2561511168	Rhodococcus erythropolis CCM2595	95.3514	95.3512	45.14	45.794
2728369692	Rhodococcus baikonurensis JCM 18801	2816332450	Rhodococcus qingshengii djl-6-2	98.5978	98.6015	46.356	45.487
2728369692	Rhodococcus baikonurensis JCM 18801	2734481946	Rhodococcus qingshengii JCM 15477	97.9323	97.9292	35.178	34.415
2728369692	Rhodococcus baikonurensis JCM 18801	2744054616	Rhodococcus qingshengii JCM 15477	98.4234	98.4269	49.575	44.63
2728369692	Rhodococcus baikonurensis JCM 18801	2617271037	Rhodococcus qingshengii TUHH-12	98.4096	98.4093	48.726	43.879
2728369692	Rhodococcus baikonurensis JCM 18801	2565956761	Rhodococcus qingshengii BKS 20-40	98.5239	98.5237	47.078	46.98
2728369692	Rhodococcus baikonurensis JCM 18801	2816332270	Rhodococcus qingshengii CS98	98.6224	98.6226	47.286	45.82
2728369692	Rhodococcus baikonurensis JCM 18801	2690315933	Rhodococcus qingshengii CW25	98.7287	98.7289	46.874	47.268

2728369692	Rhodococcus baikonurensis JCM 18801	2724679739	Rhodococcus sp. 1139	98.3886	98.271	46.904	43.928
2728369692	Rhodococcus baikonurensis JCM 18801	2724679741	Rhodococcus sp. 1163	74.0087	74.0257	13.35	19.58
2728369692	Rhodococcus baikonurensis JCM 18801	2724679742	Rhodococcus sp. 1168	74.0415	74.069	13.576	17.702
2734481946	Rhodococcus qingshengii JCM 15477	2728369692	Rhodococcus baikonurensis JCM 18801	97.9292	97.9323	34.415	35.178
2734481946	Rhodococcus qingshengii JCM 15477	2802429286	Rhodococcus enclensis NIO-1009	97.9494	97.9509	30.937	27.67
2734481946	Rhodococcus qingshengii JCM 15477	2615840625	Rhodococcus enclensis NIO-1009	97.9477	97.9491	30.889	27.653
2734481946	Rhodococcus qingshengii JCM 15477	2816332322	Rhodococcus erythropolis 1159	94.8366	94.8304	29.582	27.901
2734481946	Rhodococcus qingshengii JCM 15477	2561511168	Rhodococcus erythropolis CCM2595	95.079	95.0755	28.672	29.753
2734481946	Rhodococcus qingshengii JCM 15477	2816332450	Rhodococcus qingshengii djl-6-2	98.1908	98.1945	29.771	29.794
2734481946	Rhodococcus qingshengii JCM 15477	2744054616	Rhodococcus qingshengii JCM 15477	99.3519	99.3541	35.641	32.807
2734481946	Rhodococcus qingshengii JCM 15477	2617271037	Rhodococcus qingshengii TUHH-12	97.8279	97.8302	32.16	29.603
2734481946	Rhodococcus qingshengii JCM 15477	2565956761	Rhodococcus qingshengii BKS 20-40	98.0571	98.0526	29.949	30.585
2734481946	Rhodococcus qingshengii JCM 15477	2816332270	Rhodococcus qingshengii CS98	98.2567	98.2552	29.663	29.398
2734481946	Rhodococcus qingshengii JCM 15477	2690315933	Rhodococcus qingshengii CW25	98.2238	98.2298	29.883	30.791
2734481946	Rhodococcus qingshengii JCM 15477	2724679739	Rhodococcus sp. 1139	97.9259	97.9077	29.811	28.494
2734481946	Rhodococcus qingshengii JCM 15477	2724679741	Rhodococcus sp. 1163	73.7784	73.8442	7.997	12.15
2734481946	Rhodococcus qingshengii JCM 15477	2724679742	Rhodococcus sp. 1168	73.5973	73.604	8.364	11.117
2744054616	Rhodococcus qingshengii JCM 15477	2565956761	Rhodococcus qingshengii BKS 20-40	98.7477	98.7477	82.017	90.989
2744054616	Rhodococcus qingshengii JCM 15477	2615840625	Rhodococcus enclensis NIO-1009	98.6182	98.6203	85.369	83.119
2744054616	Rhodococcus qingshengii JCM 15477	2816332270	Rhodococcus qingshengii CS98	98.8354	98.8358	82.623	88.884
2744054616	Rhodococcus qingshengii JCM 15477	2816332322	Rhodococcus erythropolis 1159	95.4765	95.4741	81.827	83.863
2744054616	Rhodococcus qingshengii JCM 15477	2690315933	Rhodococcus qingshengii CW25	98.8788	98.8823	82.655	92.433
2744054616	Rhodococcus qingshengii JCM 15477	2728369692	Rhodococcus baikonurensis JCM 18801	98.4269	98.4234	44.63	49.575
2744054616	Rhodococcus qingshengii JCM 15477	2734481946	Rhodococcus qingshengii JCM 15477	99.3541	99.3519	32.807	35.641
2744054616	Rhodococcus qingshengii JCM 15477	2724679741	Rhodococcus sp. 1163	73.6725	73.7022	27.156	44.18
2744054616	Rhodococcus qingshengii JCM 15477	2724679742	Rhodococcus sp. 1168	73.5464	73.5488	27.71	39.778
2802429286	Rhodococcus enclensis NIO-1009	2561511168	Rhodococcus erythropolis CCM2595	95.5564	95.558	77.786	90.221
2802429286	Rhodococcus enclensis NIO-1009	2565956761	Rhodococcus qingshengii BKS 20-40	98.7426	98.752	80.61	91.955
2802429286	Rhodococcus enclensis NIO-1009	2615840625	Rhodococcus enclensis NIO-1009	100	100	99.879	99.971
2802429286	Rhodococcus enclensis NIO-1009	2744054616	Rhodococcus qingshengii JCM 15477	98.6207	98.6186	83.1	85.542
2802429286	Rhodococcus enclensis NIO-1009	2816332270	Rhodococcus qingshengii CS98	98.7303	98.7304	78.879	87.412

2802429286	Rhodococcus enclensis NIO-1009	2816332322	Rhodococcus erythropolis 1159	95.3597	95.3552	79.666	84.013
2802429286	Rhodococcus enclensis NIO-1009	2690315933	Rhodococcus qingshengii CW25	98.7531	98.7659	78.687	90.679
2802429286	Rhodococcus enclensis NIO-1009	2728369692	Rhodococcus baikonurensis JCM 18801	98.2328	98.2312	43.095	49.21
2802429286	Rhodococcus enclensis NIO-1009	2734481946	Rhodococcus qingshengii JCM 15477	97.9509	97.9494	27.67	30.937
2802429286	Rhodococcus enclensis NIO-1009	2724679741	Rhodococcus sp. 1163	73.6233	73.6356	26.34	44.141
2802429286	Rhodococcus enclensis NIO-1009	2724679742	Rhodococcus sp. 1168	73.5929	73.6221	26.928	39.88
2816332322	Rhodococcus erythropolis 1159	2565956761	Rhodococcus qingshengii BKS 20-40	95.5616	95.5551	82.712	89.588
2816332322	Rhodococcus erythropolis 1159	2816332270	Rhodococcus qingshengii CS98	95.5524	95.5569	81.941	86.153
2816332322	Rhodococcus erythropolis 1159	2690315933	Rhodococcus qingshengii CW25	95.5659	95.5683	81.163	88.908
2816332322	Rhodococcus erythropolis 1159	2728369692	Rhodococcus baikonurensis JCM 18801	95.2236	95.2327	43.715	47.315
2816332322	Rhodococcus erythropolis 1159	2734481946	Rhodococcus qingshengii JCM 15477	94.8304	94.8366	27.901	29.582
2816332322	Rhodococcus erythropolis 1159	2724679741	Rhodococcus sp. 1163	73.6689	73.6863	27.459	43.653
2816332322	Rhodococcus erythropolis 1159	2724679742	Rhodococcus sp. 1168	73.7185	73.7502	28.026	39.472
2816332450	Rhodococcus qingshengii djl-6-2	2561511168	Rhodococcus erythropolis CCM2595	95.5702	95.57	85.266	88.47
2816332450	Rhodococcus qingshengii djl-6-2	2565956761	Rhodococcus qingshengii BKS 20-40	98.9269	98.9257	87.981	89.574
2816332450	Rhodococcus qingshengii djl-6-2	2615840625	Rhodococcus enclensis NIO-1009	98.8419	98.8636	89.558	79.814
2816332450	Rhodococcus qingshengii djl-6-2	2617271037	Rhodococcus qingshengii TUHH-12	98.7717	98.7876	72.615	66.675
2816332450	Rhodococcus qingshengii djl-6-2	2744054616	Rhodococcus qingshengii JCM 15477	98.8272	98.8223	89.45	82.23
2816332450	Rhodococcus qingshengii djl-6-2	2802429286	Rhodococcus enclensis NIO-1009	98.842	98.8637	89.612	79.787
2816332450	Rhodococcus qingshengii djl-6-2	2816332270	Rhodococcus qingshengii CS98	98.7979	98.7973	88.081	87.092
2816332450	Rhodococcus qingshengii djl-6-2	2816332322	Rhodococcus erythropolis 1159	95.5367	95.5337	85.569	80.413
2816332450	Rhodococcus qingshengii djl-6-2	2690315933	Rhodococcus qingshengii CW25	98.8193	98.8179	87.674	90.215
2816332450	Rhodococcus qingshengii djl-6-2	2728369692	Rhodococcus baikonurensis JCM 18801	98.6015	98.5978	45.487	46.356
2816332450	Rhodococcus qingshengii djl-6-2	2734481946	Rhodococcus qingshengii JCM 15477	98.1945	98.1908	29.794	29.771
2816332450	Rhodococcus qingshengii djl-6-2	2724679741	Rhodococcus sp. 1163	73.6693	73.6951	28.966	43.31
2816332450	Rhodococcus qingshengii djl-6-2	2724679742	Rhodococcus sp. 1168	73.5287	73.533	29.499	38.916
